# A Primary Cortical Input to Hippocampus Expresses a Pathway-Specific and Endocannabinoid-Dependent Form of Long-Term Potentiation

**DOI:** 10.1523/ENEURO.0160-16.2016

**Published:** 2016-08-08

**Authors:** Weisheng Wang, Brian H. Trieu, Linda C. Palmer, Yousheng Jia, Danielle T. Pham, Kwang-Mook Jung, Carley A. Karsten, Collin B. Merrill, Ken Mackie, Christine M. Gall, Daniele Piomelli, Gary Lynch

**Affiliations:** 1 Department of Anatomy and Neurobiology, University of California, Irvine, California 92697; 2 Department of Psychological and Brain Sciences and Gill Center, Indiana University, Bloomington, Indiana 47405; 3 Department of Neurobiology and Behavior, University of California, Irvine, California 92697; 4 Department of Pharmacology, University of California, Irvine, California 92697; 5 Department of Biological Chemistry, University of California, Irvine, California 92697; 6 Drug Discovery and Development, Istituto Italiano di Tecnologia, 16163 Genoa, Italy; 7 Department of Psychiatry, University of California, Irvine, California 92697

**Keywords:** actin, cannabinoid, dentate gyrus, entorhinal cortex, olfactory discrimination, perforant path

## Abstract

The endocannabinoid 2-arachidonoyl-*sn*-glycerol (2-AG), a key modulator of synaptic transmission in mammalian brain, is produced in dendritic spines and then crosses the synaptic junction to depress neurotransmitter release. Here we report that 2-AG-dependent retrograde signaling also mediates an enduring enhancement of glutamate release, as assessed with independent tests, in the lateral perforant path (LPP), one of two cortical inputs to the granule cells of the dentate gyrus. Induction of this form of long-term potentiation (LTP) involved two types of glutamate receptors, changes in postsynaptic calcium, and the postsynaptic enzyme that synthesizes 2-AG. Stochastic optical reconstruction microscopy confirmed that CB_1_ cannabinoid receptors are localized presynaptically to LPP terminals, while the inhibition or knockout of the receptors eliminated LPP-LTP. Suppressing the enzyme that degrades 2-AG dramatically enhanced LPP potentiation, while overexpressing it produced the opposite effect. Priming with a CB_1_ agonist markedly reduced the threshold for LTP. Latrunculin A, which prevents actin polymerization, blocked LPP-LTP when applied extracellularly but had no effect when infused postsynaptically into granule cells, indicating that critical actin remodeling resides in the presynaptic compartment. Importantly, there was no evidence for the LPP form of potentiation in the Schaffer-commissural innervation of field CA1 or in the medial perforant path. Peripheral injections of compounds that block or enhance LPP-LTP had corresponding effects on the formation of long-term memory for cues conveyed to the dentate gyrus by the LPP. Together, these results indicate that the encoding of information carried by a principal hippocampal afferent involves an unusual, regionally differentiated form of plasticity.

## Significance Statement

A substantial literature provides a detailed description of the postsynaptic mechanisms underlying memory-related long-term potentiation (LTP) in hippocampal field CA1 and elsewhere in the cortical telencephalon. The present article introduces a different, presynaptic form of LTP localized to one of the two principal cortical inputs to hippocampus, the lateral perforant path. This projection conveys information used in the generation of episodic memory, a key element of human cognition. Intriguingly, an endocannabinoid serves as the retrograde (postsynaptic-to-presynaptic) messenger that is required for the induction of the novel plasticity effect, an observation possibly related to the influence of cannabinoid drugs on orderly thought. Finally, it is proposed that the regionally restricted version of plasticity described here enables differential encoding between hippocampal inputs.

## Introduction

Marijuana alters human cognition in profound and complex ways (Tart, 1970; Iversen, 2008). This effect is thought to reflect the interaction of its main psychoactive constituent, Δ^9^-tetrahydrocannabinol, with a modulatory system present in the brain that includes two lipid-derived neurotransmitters—2-arachidonoyl-*sn*-glycerol (2-AG) and anandamide—and their attending G-protein-coupled cannabinoid receptors (Castillo et al., 2012). 2-AG, the most abundant of these endocannabinoids (Piomelli, 2014), is produced through the activation of metabotropic glutamate 5 receptor (mGluR5) in dendritic spines (Katona et al., 2006; Jung et al., 2012a) at excitatory synapses throughout the CNS. By contrast, CB_1_ cannabinoid receptors, which mediate the majority of the synaptic actions of 2-AG, are concentrated in axon terminals (Katona et al., 2006; Uchigashima et al., 2011; Castillo et al., 2012). This morphological segregation, along with the ability of CB_1_ to depress calcium channel activity and neurotransmitter release, has led to broad acceptance of the idea that spine-derived 2-AG provides a retrograde signal that modulates release during heightened postsynaptic activity (Kano et al., 2009).

A local mechanism for transiently adjusting the strength of excitatory and inhibitory transmission would be expected to have potent effects on learning-related synaptic plasticity. In accord with this, there is a sizable literature showing that manipulating endocannabinoid signaling affects the induction of hippocampal long-term potentiation (LTP), but with conflicting results: several studies describe positive effects on potentiation (for discussion, see Carlson et al., 2002; Lin et al., 2011); while others found a negative influence (for review, see Bohme et al., 2000; Sugaya et al., 2013).

The present studies began with questions of whether and how endocannabinoids contribute to the production of LTP in the lateral perforant path (LPP), one of two cortical inputs to hippocampus (Witter, 1993; Burwell, 2000; Witter et al., 2000; van Groen et al., 2003; Amaral and Lavenex, 2007). Mechanisms at this connection are of particular interest as recent work has linked the LPP to the formation of episodic memory in rodents (Wilson et al., 2013) and humans (Reagh and Yassa, 2014), and thus to a fundamental component of cognition. Related to this, past work established that LPP-LTP is NMDA receptor (NMDAR) dependent (Hanse and Gustafsson, 1992) and is strongly modulated by local opioids (Bramham et al., 1988; Do et al., 2002). However, it is not known whether LTP in this system involves the same cellular mechanisms for stabilization and expression of the potentiated state that have been described in detail for Schaffer-commissural (S-C) afferents of field CA1 (Lynch and Gall, 2013; Granger and Nicoll, 2014). The present results demonstrate that the substrates for LPP potentiation are indeed different than at other sites in hippocampus. Data obtained with a broad array of manipulations support the conclusion that the endocannabinoid 2-AG, acting on presynaptic CB_1_ receptors during brief bursts of high-frequency afferent stimulation, elicits a lasting increase in the evoked release of glutamate from LPP terminals. This 2-AG-dependent LTP was unaffected by the suppression of GABAergic transmission and was not detected at other sites in hippocampus. In all, the results describe an unusual, pathway-specific form of LTP that may be critically involved in the production of orderly thought.

## Materials and Methods

All studies were conducted using adult rats and mice, as detailed below, and in accordance with the National Institutes of Health *Guide for the Care and Use of Laboratory Animals* and institutionally approved protocols.

### Hippocampal slices and extracellular field recordings

Studies used 5- to 8-week-old male rats (Sprague Dawley) and mice, including transgenic mice overexpressing monoacylglycerol lipase (MGL; Jung et al., 2012b) or lacking expression of the CB_1_ receptor (Deng et al., 2015); mice with genetic manipulations were evaluated in comparison with background strain-matched wild-type (WT) mice (2–3 months old, male). Acute hippocampal slices maintained in an ACSF bath in an interface recording chamber were used for electrophysiological extracellular field potential analyses. For slice preparation, animals were killed by decapitation under deep isoflurane anesthesia, and the brain was quickly submerged into oxygenated, ice-cold, high-magnesium ACSF containing the following (in mm): 124 NaCl, 3 KCl, 1.25 KH_2_PO_4_, 5 MgSO_4_, 26 NaHC0_3_, and 10 dextrose. Using a McIlwain tissue chopper for rat and a Leica vibrating tissue slicer (Model VT1000S) for mouse, slices from the middle third of the hippocampal septotemporal axis were sectioned at a thickness of 330–400 µm, collected into oxygenated, high-magnesium ACSF, and then were transferred into an interface recording chamber (32 ± 1°C, 95% O_2/_5% CO_2_) and continuously perfused with preheated oxygenated ACSF containing the following (in mm): 124 NaCl, 3 KCl, 1.25 KH_2_PO_4_, 1.5 MgSO_4_, 26 NaHCO_3_, 2.5 CaCl_2_, and 10 dextrose at a rate of 60-70 ml/h. Experiments were initiated ∼1.5 h after slices were placed in the recording chamber.

Field EPSPs (fEPSPs) for the LPP, medial perforant path (MPP), and field CA1 S-C systems were elicited using bipolar stimulating electrodes (65 µm twisted nichrome wire) and recorded with a glass recording electrode (2 m NaCl filled; 2–3 MΩ; Trieu et al., 2015). Switching the anode from one pole to the other of the bipolar stimulation electrode substantially changes the magnitude of the elicited fEPSP, indicating minimal spread of current from the active tip (48.6 ± 8.1%, *n* = 6; *p* < 0.003, paired *t* test). To verify placement in the LPP versus MPP system, evoked responses were initially tested with paired-pulse stimuli (40 and 200 ms interpulse intervals). LPP responses show paired-pulse facilitation, and MPP responses show paired-pulse depression (Christie and Abraham, 1994). Stable baseline recordings of responses to low-frequency stimulation (single pulses delivered at 0.05 Hz with stimulation intensity was adjusted to 50–60% of the maximum spike-free fEPSP) were collected for at least 20 min prior to pharmacological manipulation or the induction of LTP, which entailed the following: (1) for the LPP, two 100 Hz trains, each lasting 1 s and separated by 1 min with stimulus duration and intensity at 2× and 1.5× baseline levels, respectively; (2) for the MPP, three 100 Hz trains, 500 ms each, and separated by 50 s, delivered at twice the duration of baseline stimulation and in the presence of picrotoxin (PTX; Hanse and Gustafsson, 1992); and (3) for S-C projections, a single train of 10 theta bursts (TBS: 100 Hz bursts of four pulses, separated by 200 ms, delivered at baseline stimulus intensity; Larson et al., 1995).

Initial fEPSP slopes and amplitudes were measured from digitized traces (NACGather 2.0, Theta Burst Corp.) and normalized to mean responses over the last 5 min of the baseline period. Plots of electrophysiological measures (fEPSPs and whole-cell recordings) show group mean ± SEM values. The magnitude of LTP was assessed by measures of fEPSP slope for the 5 min period from 55 to 60 min after inducing stimulation, relative to mean responses during the last 5 min of baseline recordings, unless otherwise noted. Paired-pulse facilitation (PPF; i.e., the percentage increase in the second evoked response relative to the first in the pair) was assessed using a 40 ms interval between pulses. Slices in which the initial PPF, during the baseline period, was >75% (8.5% of cases) were not included in the analysis. For all electrophysiological studies, *N* values given in captions are for the total number of slices from at least three animals per group.

For hippocampal slice field recording studies, compounds were introduced to the ACSF bath (6 ml/h) via an independent perfusion line using a syringe pump; the period of infusion is indicated with a horizontal gray line in figures. The following reagents and treatment concentrations were used: NMDAR antagonist dl-APV sodium salt (100 µm), latrunculin A (Lat-A; 500 nm), and CB_1_ antagonist SR141716A (5 µm for field and 25 µm for whole-cell experiments; all from Tocris Bioscience); CB_1_ agonist WIN55,212-2 (WIN) mesylate (5 µm), CB_1_ inverse agonist AM251 (5 µm), and tetrahydrolipstatin (THL; 10 µm; Orlistat; all from R&D Systems); JZL184 (1 µm; from RTI International); PTX (5–100 µm as noted; from Sigma-Aldrich), URB597 (1 µm; from Italian Institute of Technology), WWL70 (10 µm; from Cayman Chemical), and 2-methyl-6-(phenylethynyl)pyridine hydrochloride (MPEP; 40 µm; donated by the FRAXA Research Foundation). MPEP, AP5, and (*RS*)-3,5-dihydrophenylglycine were dissolved in water. Other compounds were dissolved in 100% dimethylsulfoxide (DMSO) or ethanol and diluted in ACSF to a final concentration of ≤0.1% diluent in the ACSF bath; the one exception was WWL70, for which the final bath DMSO concentration was 0.5%. The effects of AM251, SR141716A, WIN55,121-2, JZL184, URB597, and MPEP generated the same results with and without PTX in the ACSF bath.

Past experience indicates that consistency across experiments is best achieved by having a single investigator conduct all phases of a given slice study. It is very difficult to fully blind such experiments, but a number of steps were taken to avoid bias. These included between-group comparisons of the following pretreatment measures: stimulation currents; waveforms of synaptic potentials; input/output curves; and paired-pulse facilitation. There was detailed analysis of the raw data along with each stage of its processing by a second investigator. Moreover, the effects of experimental treatments were tested on slices from the same animal evaluated in parallel on separate chambers, and a number of points were tested by two researchers. In the case of mutants, it was possible to test groups blindly when differences in physical characteristics or behavior, or markers for gene cohort, were absent.

### Whole-cell recordings

Hippocampal slices were prepared on the horizontal plane at a thickness of 370 µm from 3- to 4-week-old rats with a Leica vibrating tissue slicer. Slices were placed in a submerged recording chamber and continuously perfused at 2–3 ml/min with oxygenated (95% O_2_/5% CO_2_) at 32°C. Whole-cell recordings (Axopatch 200A amplifier; Molecular Devices) were made with 4–7 MΩ recording pipettes filled with a solution containing (in mm): 130 CsMeSO_4_, 10 CsCl, 8 NaCl, 10 HEPES, 0.2 EGTA, 5 QX-314, Mg-ATP, and 0.3 Na-GTP. Osmolarity was adjusted to 290–295 mOsm and pH buffered at 7.25. Bipolar stimulating electrodes were placed in the outer molecular layer to stimulate the LPP. EPSCs were recorded by clamping the granule cell at −70 mV in the presence of 50 µm PTX (Jia et al., 2010). LTP was induced using a pairing protocol of 2 Hz stimulation for 75 s at a holding potential of −10 mV, after recording a stable 10 min baseline. The LPP-mediated PPF was measured before and after LTP induction by the application of paired-pulse stimulation at an interval of 100 ms. To assess the NMDAR- and AMPA receptor (AMPAR)-mediated EPSC ratio, cells were held at +40 mV, and AMPAR-mediated currents isolated with the selective NMDAR antagonist dl-APV. The NMDAR-mediated currents were then determined by subtracting the AMPAR-mediated currents from the total EPSC. To specifically study postsynaptic effects, 400 nm latrunculin A or 10 mm BAPTA was applied postsynaptically via intracellular infusion through the patch pipette applied to individual granule or pyramidal cells.

### Lipid quantification and activity assays

Levels of 2-AG, oleoylethanolamide (OEA), and stearic acid were determined using published liquid chromatography/mass spectrometry (LC/MS) methods. Immediately after behavioral testing, dentate gyrus (DG) and CA1 fields were dissected and snap frozen, or punches were taken of dentate gyrus and auditory cortex from fast-frozen, cryostat-mounted brains. From methanol homogenates, lipids were extracted with chloroform, and further fractionated by open-bed silica gel column chromatography. The eluates were dried under N_2_ and reconstituted in chloroform/methanol for LC/MS analyses. Measures from both types of sampling were combined after normalization to respective control regions. Activities of fatty acid amide hydrolase (FAAH) and 2-AG-hydrolase MGL were measured using published techniques (King et al., 2007; Moreno-Sanz et al., 2013). Dentate gyrus results were normalized for each animal to values for the auditory cortex or field CA1.

### Stochastic optical reconstruction microscopy imaging

For three-dimensional super-resolution Stochastic optical reconstruction microscopy (STORM) imaging, fresh-frozen brains from young adult 5- to 8-week-old male Sprague Dawley rats were sectioned and processed for dual immunofluorescence using a cocktail of primary antisera, including rabbit anti-CB_1_ (Mátyás et al., 2006) with mouse anti-SYN (MAB5258 clone Sy38, Millipore) or mouse anti-postsynaptic density-95 (PSD-95; MA1-045 clone 6G6-1C9, Thermo Scientific) and secondary antisera conjugated to photoswitchable dyes (Alexa Fluor 647 and Atto 488). Immunolabeling was visualized using a Nikon TIRF microscope equipped with a 100× objective and ANDOR iXon CCD camera; this system supports resolution of individual molecules as close together as 20 nm (Dani et al., 2010). Both channels were recorded simultaneously until ≥500,000 molecules were detected, and then Nikon NIS software was used to reconstruct the image free of nonspecific background activation. Using Python 2.7, densely labeled synapses were identified by applying a clustering algorithm (Rodriguez and Laio, 2014) to the field of molecules to filter out elements smaller than seven molecules. Coordinates for the centroid of each cluster were used to calculate distances between elements.

### Odor discrimination behavior

All behavioral sessions were video recorded by overhead camera, and behavioral analyses were conducted from the videos by individuals blind to treatment. Well handled male Long–Evans rats (6–10 weeks old) were kept on a reverse 12 h light/dark cycle, maintained at 90% normal body weight, and handled for six sessions, two sessions per day, prior to odor discrimination training (Martens et al., 2013). The test apparatus consists of a 15 × 28 × 15 inch white acrylic box, resting on a chromed wire grid with holes cut to accept 2-inch-diameter plastic or glass “digging cups” to hold sand for behavioral testing. For odor discrimination training, rats were trained to distinguish between unique odors presented in pairs, with one odor per pair rewarded. Odorants were finely pulverized household spices, mixed with clean dry sand at a ratio of 1 g of odorant to 100 g of sand (odorants included anise, basil, cinnamon, coffee, cocoa, coriander, cumin, dillweed, fenugreek, ginger, lavender flower, oregano, peppermint, rose petal, rosemary, and sage). Sand cups were filled with the mixture with one cup baited, and the rat was allowed to dig; if the rat chose the wrong cup or did not dig before 30 s, it was removed until the next trial; if it chose the correct cup, it was allowed to consume the half-Froot Loop treat and then removed until the next trial. The intertrial interval was 20–30 s; cups were replaced and positions randomized between trials. Sessions of 10 30 s trials on a given odor pair were repeated up to twice daily until rats reached a success rate of ≥80% correct before the introduction of a new odor pair. Testing of drug effects on learning novel odors commenced on the following day. For AM251 treatments, rats were given an intraperitoneal injection of AM251 (1 mg/kg) or vehicle (veh: DMSO, cremophore, ethanol, 0.9% saline, at 1:1:1:17) 1 h prior to training (10 trials) on a new odor pair. Rats were tested again 24 h later on the same pair of odors. For JZL184 treatments, rats were given an intraperitoneal injection of this compound (16 mg/kg, i.p.) or vehicle (80% polyethylene glycol 200, 20% Tween 80) 6 h prior to six training trials; rats were then killed immediately following training for neurochemical measures or were tested for retention 24 h later on the same odor pair.

### Statistics

All results are presented as mean ± SEM values. Statistical significance (*p* < 0.05) was evaluated using one- or two-tailed Student’s *t* test, the nonparametric Mann–Whitney or Wilcoxon signed rank test (for comparison of two groups), and the two-way ANOVA, as indicated (Prism, GraphPad). In graphs, asterisks denote the level of significance (*p < 0.05; **p < 0.01; ***p < 0.001).

## Results

### 2-AG-dependent LTP in the lateral perforant path

**Figure 1.**
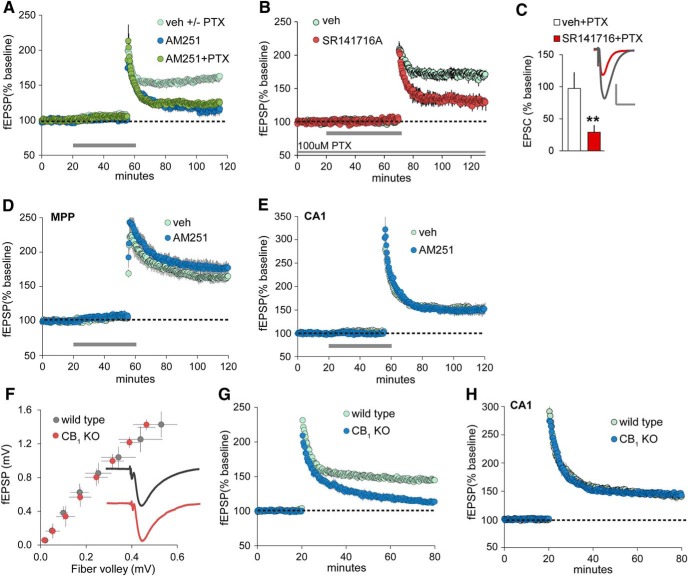
LPP-LTP is endocannabinoid and CB_1_ dependent. ***A***, The CB_1_ inverse agonist AM251 (5 µm, at horizontal bar) disrupts LPP-LTP with or without PTX (10 µm) in the ACSF bath. Veh-treated slices included those tested with (+) and without (−) PTX present. Potentiation, assessed 55–60 min after high-frequency stimulation, was greatly reduced in both AM251 groups (*p* < 0.0004, −PTX; *p* < 0.001, +PTX; *n* = 5 each) relative to veh. ***B***, Perfusion of the CB_1_ antagonist SR141716A (5 µm) in the presence of 100 µm PTX significantly attenuated LPP-LTP, as assessed with field recordings (*p* = 0.0004, two-tailed *t* test for percentage of LTP in veh vs SR141716A). ***C***, CB_1_ antagonist SR141716A also significantly reduced the percentage of LPP potentiation as assessed 25 min postinduction using voltage-clamp recordings of granule cells in slices treated with veh (*n* = 8) or 25 µm SR141716A (*n* = 6; **p < 0.005, two-tailed *t* test vs veh+PTX): traces show EPSCs collected before and 30 min after induction for veh slices. Calibration: 100 pA, 50 ms. ***D***, ***E***, AM251 did not reduce LTP of the MPP (***D***) or the S-C afferents of field CA1 (***E***; *n* = 5/group). ***F***, Relationship of the peak amplitude of the fiber volley to the fEPSP slope (input/output curves) in the outer molecular layer of the DG with LPP stimulation in WT and CB_1_ KO mice: traces are averaged group responses after normalization of mean fEPSPs for individual slices to their peak amplitude. Group fEPSPs for WT and CB_1_ KO slices were indistinguishable. ***G***, LTP in the LPP was severely impaired in CB_1_ KO mice (*p* < 0.0001; *n* = 10/group). ***H***, LTP did not differ between CB_1_ KOs and WTs in field CA1 (*p* = 0.89, *t*_(10)_ = 0.16: *n* = 6/group). All panels show results from field recordings (fEPSPs), except for the bar graph in ***C***, which shows the results of whole-cell, voltage-clamp recordings.

High-frequency stimulation (HFS) of the LPP in acute rat hippocampal slices elicited enduring LTP at LPP synapses with DG granule cells. In studies using field recordings, this potentiation was prevented by bath application of the CB_1_ inverse agonist AM251 (5 µm; Fig. 1A). Adding PTX (10 µm) to the ACSF perfusate did not measurably affect the magnitude of LPP-LTP in slices treated with veh (without PTX, 59.1 ± 8.6%; with PTX, 62.2 ± 4.1%; *p* > 0.75, two-tailed *t* test) or with AM251 (*p* < 0.004, without PTX; *p* < 0.001, with PTX, two-tailed tests; Fig. 1A). To confirm that endocannabinoids do not promote LPP-LTP via actions on fast inhibitory transmission, we repeated the field recording experiment using the CB_1_ antagonist SR141716A (5 µm; Khaspekov et al., 2004) applied in the presence of 100 µm PTX: LTP was greatly reduced by the antagonist (percentage of LTP: vehicle plus PTX, 70.3 ± 4.9%; SR141716A plus PTX, 28.7 ± 5.0%; *p* = 0.0004, two-tailed *t* test; Fig. 1B). Finally, we evaluated SR141716A effects on LPP potentiation using whole-cell recordings and in the presence of 50 µm PTX, conditions in which we could verify that IPSCs were fully eliminated. LPP-LTP was again markedly reduced: percentage of LTP at 25 min postinduction was 97.4 ± 24.9% with vehicle plus PTX and 29.1 ± 10.8% with SR141716A plus PTX present (*p* < 0.005, two-tailed *t* test; Fig. 1C).

Initial potentiation (during the first 2 min after HFS) in the field potential studies was not reliably affected by AM251 (veh, 98.1 ± 7.7%; AM251, 88.3 ± 10.0%; *p* > 0.40). We conclude from this that CB_1_ receptors contribute to the stabilization, as opposed to the induction, of the potentiated state of LPP synapses. CB_1_-dependent potentiation appeared to be restricted to LPP synapses because AM251 had no effect on LTP at the adjacent MPP synapses (62.8 ± 6.9% for veh and 76.2 ± 8.5% for AM251; 5 µm PTX in bath, both groups; Fig. 1D) or for S-C projections to field CA1 (53.5 ± 6.6% for veh and 50.5 ± 11.2% for AM251; Fig. 1E).

The obligatory role of CB_1_ in LPP-LTP was confirmed in experiments with hippocampal slices from CB_1_ knock-out (KO) mice (Deng et al., 2015). First, we tested whether the mutation changes basic response characteristics in the LPP using field recordings. The input/output relationship (number of axons stimulated—“fiber volley”—vs size of the evoked response) did not differ between WT and KO mice. The averaged fEPSP waveform for the KOs was also comparable to that in the WTs (Fig. 1F). The mean time constant for the rising phase (rise tau) of the response (from start to 80% maximum) was 2.14 ± 0.44 ms for WTs and 1.94 ± 0.25 ms for CB_1_ KOs, and the time constant for the decay phase (decay tau) of the fEPSP was also not detectably different between genotypes (WT, 7.89 ± 1.46 ms; CB_1_ KO, 6.03 ± 2.62 ms). Despite the apparent normality of baseline evoked potentials, high-frequency stimulation failed to induce stable LTP at LPP synapses in CB_1_ KOs (WTs, 43.3 ± 3.1; CB_1_ KOs, 13.4 ± 2.9; *p* < 0.0001; Fig. 1G), although reliable potentiation was produced at S-C synapses in field CA1 in these mice (42.1 ± 6.2% for WTs and 43.7 ± 1.7% for KOs; Fig. 1H). A recent study (Gómez-Gonzalo et al., 2015) reported CB_1_-dependent, heterosynaptic facilitation in field CA1. However, in agreement with earlier work (Dunwiddie and Lynch, 1978; Wigström and Gustafsson, 1986; Lovinger and Routtenberg, 1988; Abraham et al., 1994), the induction of LTP in the present experiments did not increase responses to neighboring inputs in the S-C innervation of field CA1.

**Figure 2.**
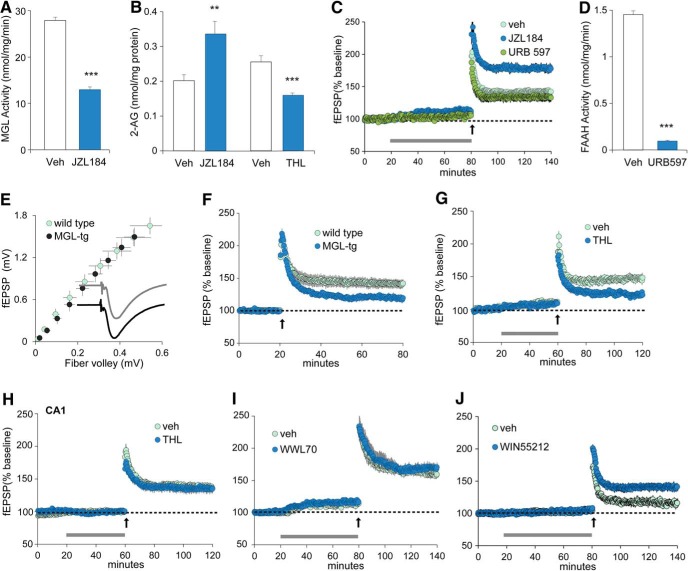
Lateral perforant path LTP depends upon the endocannabinoid 2-AG. ***A***, A 60 min treatment of acute hippocampal slices with JZL184 (1 µm) reduced by 50% activity of the 2-AG degradative enzyme MGL (***p < 0.001 vs veh, *t* test; *n* = 9 each). ***B***, Hippocampal slice levels of 2-AG were increased by JZL184 (1 µm; **p < 0.01; *n* = 10 for veh and JZL184) but decreased by perfusion of THL (10 µm), an inhibitor of the 2-AG synthesizing enzyme DGL-α (***p < 0.001; veh, *n* = 4; THL, *n* = 5). ***C***, As determined with fEPSP recordings, the magnitude of LPP potentiation was increased by JZL184 (1 µm) but was not affected by URB597 (1 µm), an inhibitor of FAAH. ***D***, URB597 (1 µm) perfusion for 60 min reduced hippocampal slice FAAH activity (***p < 0.001, *n* = 10 each). ***E***, LPP input/output curves were comparable for wild-type and MGL-overexpressing transgenics (MGL-tg) as were waveforms for averaged fEPSPs. ***F***, LPP-LTP was reduced in MGL-tg vs wild-type mice (*p* = 0.016; WT, *n* = 14; MGL-tg, *n* = 17). ***G***, ***H***, THL (10 µm) perfused for 60 min significantly reduced LPP-LTP (***G***; *n* = 8/group) but not in S-C projections to field CA1 (***H***; *n* = 5/group). ***I***, WWL70, an inhibitor of the postsynaptic 2-AG degradative lipase ABHD6, had no effect on LPP-LTP; the modest effect of infusion on baseline responses reflects DMSO in the vehicle. ***J***, Infusion of the CB_1_ agonist WIN55,212-2 (5 µm) for 1 h prior to, and overlapping, a brief high-frequency stimulation train (arrow) increased the magnitude of LTP relative to levels with veh treatment (*p* = 0.004; *n* = 6 for veh group, *n* = 7 for WIN group); WIN did not significantly influence baseline responses relative to those of the vehicle treatment group. In ***C*** and ***F–J***, the horizontal gray bar denotes the period of reagent perfusion; all electrophysiological analyses are from field recordings. Upward arrows indicate the time of LTP-inducing stimulation.

To identify the specific endocannabinoid involved in LTP at LPP contacts, we first tested the effects of agents that interrupt the enzyme-mediated degradation of either anandamide or 2-AG, the two major endocannabinoids in brain. JZL184 (1 µm), an inhibitor of the 2-AG-degrading lipase MGL (Long et al., 2009), which is localized to presynaptic elements in hippocampus (Gulyas et al., 2004), was verified to both suppress MGL activity (Fig. 2A) and, as expected from this, to increase 2-AG levels (Fig. 2B) in hippocampal slices. The infusion of JZL184, initiated 60 min before HFS, markedly enhanced LPP-LTP (43.2 ± 4.0% for veh; 78.3 ± 5.9% for JZL184; *p* = 0.001, *t* test, two-tailed). In contrast, LPP potentiation was not increased by URB597 (1 µm; 42.3 ± 3.2% for veh; 34.1 ± 6.2% for URB597; *p* > 0.25; *n* = 8 for veh, URB597; *n* = 5 for JZL184; Fig. 2C), which inhibits the anandamide-degrading enzyme FAAH (Fig. 2D). Thus, the magnitude of LPP potentiation was increased by a manipulation (JZL184) that increases 2-AG levels but not by one (URB597) that elevates anandamide.

We further tested the role of 2-AG in LPP-LTP using transgenic (tg) mice that selectively overexpress MGL in forebrain neurons; 2-AG levels in brain are reduced by ∼50% in these mice (Jung et al., 2012b). Input/output curves for the LPP were comparable for MGL-tg versus WT mice (*p* > 0.50, repeated measure (RM) ANOVA), and mean waveforms for the groups were superimposable (Fig. 2E). Quantitative comparisons of mean rise and decay time constants confirmed that the elevated 2-AG levels in the mutants had no measurable effects on baseline excitatory transmission (rise tau: WT, 2.29 ± 0.22 ms; MGL-tg, 2.16 ± 0.24 ms; decay tau: WT, 10.66 ± 0.47 ms, MGL-tg, 10.78 ± 1.13 ms). However, LPP-LTP was significantly reduced in slices from MGL-tg mice (WT, 41.2 ± 6.8%; MGL-tg, 19.4 ± 4.7%; *p* = 0.016; Fig. 2F). Consistent with these results, treatment with THL (10 µm), an inhibitor of the 2-AG-synthetic enzyme diacylglycerol lipase-α (DGL-α), both substantially lowered 2-AG levels in hippocampal slices (Fig. 2B) and significantly reduced LPP-LTP (veh, 47.4 ± 5.6%; THL, 22.9 ± 2.5%; *p* < 0.002, two-tailed *t* test; Fig. 2G). This treatment did not have reliable effects on TB-induced LTP of S-C projections in field CA1 (veh, 43.3 ± 4.0%; THL, 34.4 ± 6.6%; *p* > 0.20; Fig. 2H). Finally, inhibiting the postsynaptic 2-AG-hydrolyzing lipase, αβ-hydrolase domain-6 (Naydenov et al., 2014) with WWL70 (10 µm) did not affect LPP-LTP (veh, 41.5 ± 2.9%; WWL70, 44.9 ± 4.5%; *n* = 6 each; Fig. 2I).

Together, the above results obtained with agents or genetic manipulations that enhance or depress 2-AG signaling, support the conclusion that LTP in the LPP depends upon released 2-AG acting on CB_1_ receptors and mechanisms that do not entail suppression of GABAergic inhibition. These same studies did not detect the contributions of endocannabinoid or, more specifically, 2-AG involvement in activity-induced potentiation of the MPP or the S-C innervation of field CA1.

### The threshold for LPP-LTP is markedly reduced by stimulation of CB_1_ receptors

The above conclusion was further tested by asking whether direct stimulation of CB_1_ receptors promotes the induction of LTP in the LPP. We first established a stimulation paradigm (100 Hz for 200 ms) that is near the threshold for producing a measurable degree of stable potentiation. This stimulation protocol was then applied to slices in the presence of vehicle or the CB_1_ agonist WIN at a concentration (5 µm) widely used in physiological and biochemical experiments (Schmitz et al., 2016); the effects of the agonist were evaluated with and without 100 µm PTX present. There was no evident effect of WIN on baseline responses, but LPP potentiation was clearly enhanced: absent PTX, the mean percentage potentiation at 60 min in vehicle-treated slices was 12.3 ± 4.2% (*n* = 6), a value well below that obtained with full-length trains of high-frequency stimulation, and 35.3 ± 4.7% (*n* = 7) for the WIN treatment group (*p* = 0.004; Fig. 2J). In the presence of PTX, the percentages of potentiation were 16.67 ± 5.87% (*n* = 6) and 43.43 ± 5.33% (*n* = 7) for vehicle and WIN treatment groups, respectively (*p* = 0.006). These results stand in marked contrast to those reported for S-C inputs of field CA1 that describe a clear inhibition of LTP induction with WIN infusion (Paton et al., 1998). The differential effects of WIN on stimulation-induced LTP constitute additional evidence for the marked difference in the substrates for potentiation between the LPP and field CA1 S-C systems, and support the conclusion that the triggering mechanisms for LTP in the former system, but not the latter system, involve endocannabinoid signaling.

### LTP in the LPP is induced postsynaptically

**Figure 3.**
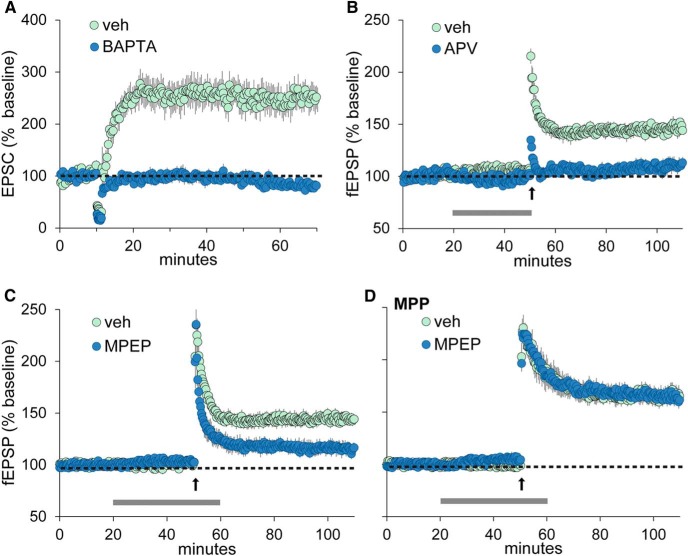
Endocannabinoid-dependent LTP is blocked by buffering postsynaptic calcium and by glutamate receptor antagonists. ***A***, Whole-cell recordings of EPSCs elicited by single-pulse stimulation of the LPP were collected from granule cells with voltage-clamp electrodes containing veh (*n* = 7) or the calcium-chelating agent BAPTA (*n* = 10). Repetitive stimulation, with the membrane potential held at 0 mV, produced robust LTP in vehicle cases but no potentiation in BAPTA experiments. ***B***, Perfusion of NMDAR antagonist APV (100 µm) blocked LPP-LTP in studies using field potentials (*p* < 0.0004; *n* = 5/group). ***C***, ***D***, In field recording experiments, the mGluR5 antagonist MPEP (40 µm) disrupted LPP-LTP (*p* < 0.0006; *n* ≥ 9/group; ***C***) but had no effect on potentiation of the MPP (*p* > 0.18, *t*_(10)_ = 1.5, *n* = 6/group; ***D***). PTX (10 µm) was included in LPP experiments summarized in ***A–C***; horizontal bars indicate periods of reagent perfusion. Upward arrows in ***B–D*** indicate the application of HFS.

2-AG signaling at glutamatergic synapses starts with mGluR5-dependent activation of DGL-α, which converts the membrane-bound 2-AG precursor 1-acyl-2-arachidonoyl-*sn*-glycerol into diffusible 2-AG (Bisogno et al., 2003; Jung et al., 2007). DGL-α requires calcium for activity and is localized to dendritic spines, where it forms a multimolecular complex with mGluR5 (Jung et al., 2005, 2012a). Consistent with this model, we found that HFS-induced LPP-LTP is eliminated by voltage clamping granule cells with electrodes containing the calcium-buffering agent BAPTA, leading to intracellular infusion of the compound (percentage LTP: for veh, 148.0 ± 25.2%; for BAPTA, −14.4 ± 6.0%; *p* < 0.0006, two-tailed *t* test; Fig. 3A) or by bath perfusion with the NMDAR antagonist APV (percentage LTP: for veh, 47.2 ± 4.1%; for APV, 11.1 ± 4.6%; *p* < 0.0004, two-tailed *t* test; Fig. 3B). The mGluR5 antagonist MPEP (40 µm) also markedly reduced LPP-LTP (veh, 44.3 ± 4.3%; MPEP, 15.3 ± 5.4%; *p* < 0.0006; Fig. 3C) without significantly affecting potentiation at MPP synapses (veh, 66.7 ± 6.4%; MPEP, 64.0 ± 5.1%; *p* = 0.18; Fig. 3D) or in field CA1 (veh, 52.5 ± 7.1%; MPEP, 45.9 ± 3.6%; *p* = 0.27, *n* = 6/group; data not shown). It is interesting that postsynaptic calcium buffering or NMDAR antagonism blocked initial potentiation (∼2 min after induction), while MPEP (or AM251; see above) did not, even though all four manipulations blocked the production of stable LPP-LTP. These observations suggest that high-frequency stimulation generates rapid and delayed stages of potentiation in the LPP, with the former initiated by NMDAR-induced increases in postsynaptic calcium, and the latter by released 2-AG transmission. The initiation of the second, stable phase of response facilitation is clearly dependent upon first-stage events.

It is noteworthy that the above manipulations did not affect the inward currents or depolarization of granule cell dendrites produced by high-frequency stimulation of the LPP. Past studies have shown that seconds-long depolarization of neurons via clamp electrodes depresses release from their excitatory and inhibitory afferents (depolarization-induced suppression of excitation or inhibition) via CB_1_ receptors (Chiu and Castillo, 2008). These effects are reported to be absent in both the LPP and MPP (Chiu and Castillo, 2008). It was therefore not possible to test how this form of endocannabinoid signaling is affected by the induction of LPP-LTP.

### Evidence that LPP potentiation is expressed presynaptically

**Figure 4.**
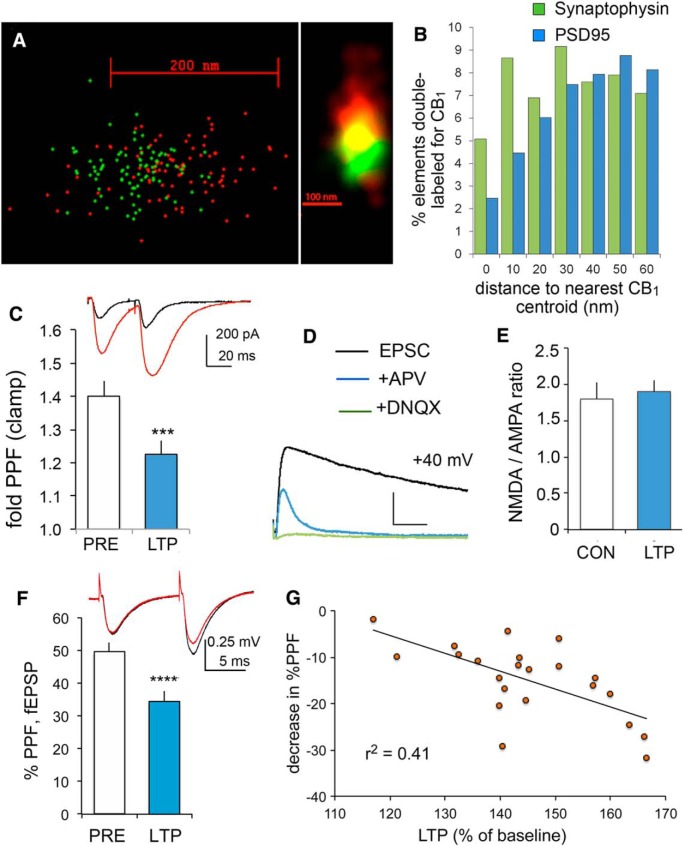
Presynaptic localization of CB_1_ receptors and expression of LPP-LTP. ***A***, Illustration of STORM images showing the overlapping distribution of CB_1_ (red) and synaptophysin (SYN; green) immunoreactivity in a terminal within the DG outer molecular layer when visualized as nanoscale localization points (left) and density blur of the same points showing the field of codistribution in yellow (right; the blur image is rotated 90°). ***B***, Histogram of nearest-neighbor distances between cluster centers of CB_1_ and either SYN^+^ or PSD-95^+^ elements. CB_1_^+^ clusters are more closely associated (<30 μm) with SYN, indicating a preferential presynaptic localization (*n* = 7 rats each). ***C***, traces, Clamp recordings from a granule cell with paired-pulse stimulation of the LPP (40 ms interval between the pulses) before (black trace) and 30 min after (red trace) induction of LTP. Calibration: 200 pA, 20 ms. The graph shows that the magnitude of LPP PPF was reduced after potentiation (*p* = 0.0003, paired two-tailed *t* test, *n* = 10). ***D***, To assess NMDAR and AMPAR currents, EPSCs were measured with the membrane potential held at +40 mV before (black line) and after (blue line) the infusion of APV; DNQX was infused at the end of the recording session (green line). Calibration: 200 pA, 20 ms. ***E***, LTP did not decrease the NMDAR/AMPAR current ratio [*p* > 0.75; control (CON), *n* = 6; LTP, *n* = 7]. ***F***, traces, Field recordings show LPP fEPSPs with paired-pulse stimulation of the LPP (40 ms between the pulses) before (black trace) and 30 min after (red trace) the induction of LTP. Bar graph shows that PPF of the fEPSP slope was reduced 30 min after inducing LPP-LTP relative to PPF during the pre-LTP baseline period (****p < 0.00001, *n* = 22;). ***G***, In LPP field recordings, the change in PPF after the induction of LTP correlated with the magnitude of LTP.

The LPP originates in the lateral entorhinal cortex and terminates in the outer molecular layer of the dentate gyrus (Witter, 1993; van Groen et al., 2003; Amaral and Lavenex, 2007). We found that in this field, as in many other brain regions (Katona et al., 2006; Mátyás et al., 2007; Mackie, 2008; Uchigashima et al., 2011), a substantial number of presynaptic nerve endings contain CB_1_ receptors: dual-immunolabeling STORM microscopy experiments revealed a high degree of overlap between immunoreactivity for CB_1_ and the axon terminal marker synaptophysin (Fig. 4A). We quantified distances between centroids for CB_1_ and synaptophysin-immmunopositive (+) clusters in 36 sampling fields from seven rats and obtained a mean value of 90.1 ± 5.8 nm. The corresponding values for immunoreactivities for CB_1_ and PSD-95, which is concentrated in glutamatergic synapses, were 118.7 ± 3.0 nm (39 sampling fields; *p* < 0.00001, *t* test). There was a significant interaction effect between distance and synaptic compartment (synaptophysin vs PSD-95) when comparing the frequency of CB_1_+ clusters at different distances (RM two-way ANOVA, *p* < 0.005; Fig. 4B). In addition, colocalization analysis showed that >40% of synaptophysin+ clusters contained CB_1_ immunoreactivity (43.1 ± 4.9%). Since the excitatory entorhinal projections comprise >80% of terminations in the DG molecular layer (Amaral and Lavenex, 2007) these results confirm that CB_1_ receptors are localized in part to LPP terminals in this field.

The imaging and pharmacological results suggest that LPP-LTP could be expressed through presynaptic changes. We used PPF (Muller and Lynch, 1988) and whole-cell recordings of rat DG granule cells to test whether the effect is associated with changes in neurotransmitter release probability. Consistent with a presynaptic locus (Manabe and Nicoll, 1994), potentiation was accompanied by a substantial reduction in PPF, as occurs when release is enhanced (*p* < 0.0003, paired two-tailed *t* test; Fig. 4C). Of interest, a recent study reported a smaller (<10%) decrease in PPF after the induction of endocannabinoid-supported LTP in striatum (Cui et al., 2015). LTP was also accompanied by equivalent increases in the magnitude of synaptic currents gated by AMPA and NMDA receptor channels. For these studies, EPSCs were sampled with the membrane potential held at +40 mV before and after infusion of the NMDAR antagonist APV; the remaining (APV-insensitive) portion of the EPSC was due to AMPAR-gated currents, as confirmed with subsequent application of the AMPAR antagonist DNQX (Fig. 4D). Subtracting the APV-insensitive response from the pre-APV EPSC yielded the NMDAR-mediated current. The ratio of the two components of the EPSC was then calculated and found to be comparable in control slices and those tested 30 min after induction of LPP-LTP (control, 1.82 ± 0.22; LTP, 1.90 ±0.16; *p* > 0.75, *t* test; Fig. 4E). Past studies in field CA1 showed that enhancing glutamate release from terminals produces similar increases in AMPAR- and NMDAR-mediated postsynaptic responses, whereas LTP selectively enhances AMPAR-gated currents (Kauer et al., 1988; Muller and Lynch, 1988). Accordingly, the results just described support the conclusion that, in contrast to CA1, LTP in the LPP is most likely due to presynaptic changes.

Paired-pulse facilitation results comparable to those found with voltage-clamp recordings were also obtained using extracellular field potentials (49.6 ± 2.9% pre-LTP; 34.5 ± 3.0% post-LTP; *p* < 0.00001, paired *t* test; Fig. 4F). A large number of slices was tested (*n* = 22) in order to determine whether the magnitude of fEPSP LTP was negatively correlated with the percentage change in PPF, as expected if the first variable is associated with the second. The results confirmed the prediction (percentage LTP vs percentage decrease in PPF: *r* = −0.638, *p* = 0.0014, Pearson test; Fig. 4G).

It is of interest that previous work indicates that the mossy fiber output of the granule cells expresses a long-lasting form of release enhancement (Stäubli et al., 1990; Nicoll and Schmitz, 2005). However, LPP-LTP depends upon the concomitant activation of CB_1_ and NMDA receptors, both of which are dispensable for mossy fiber potentiation (Hofmann et al, 2008).

### Presynaptic actin polymerization is required for LPP-LTP

CB_1_ receptors engage G_i/_G_o_ proteins to initiate multiple signal transduction processes (Howlett and Mukhopadhyay, 2000; Keimpema et al., 2011). While the effects on calcium channel activity have been a main focus of attention, results from studies of dissociated cells indicate that CB_1_ can promote cytoskeletal reorganization: CB_1_ receptors via G-proteins activate a large number of signaling cascades, including several small GTPases (RhoA, Rac, Rap) that engage, among other targets, actin regulatory elements (He et al., 2005; Nithipatikom et al., 2012; Roland et al., 2014; Mai et al., 2015; Njoo et al., 2015) and can effect actin-dependent changes in release (Malenczyk et al., 2013). These observations suggested the possibility that cytoskeletal changes underlie enhanced glutamate release with LPP-LTP.

**Figure 5.**
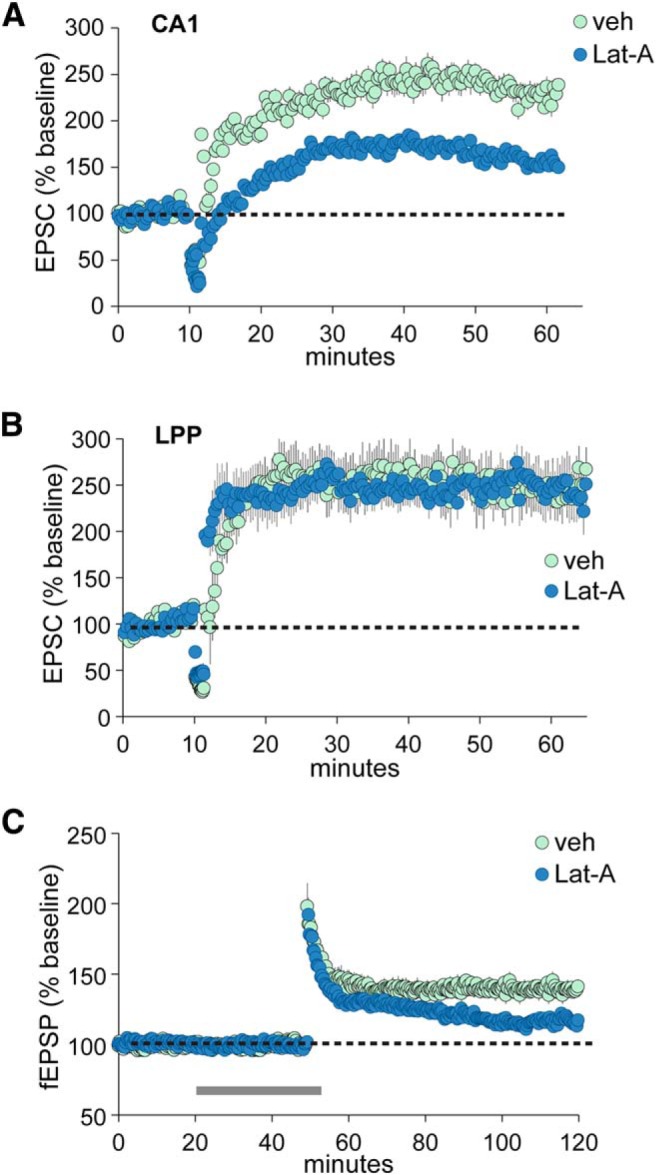
Presynaptic cytoskeletal changes are required for LPP-LTP. ***A***, Whole-cell, voltage-clamp recordings from CA1 pyramidal cells using electrodes containing Lat-A (400 nM) or veh. Baseline EPSCs were obtained at 3/min for 10 min and then repetitive stimulation applied with membrane potential held at 0 mV. Intracellular Lat-A infusion markedly reduced field CA1 LTP (*p* < 0.026, *n* ≥ 6/group). ***B***, Whole-cell recordings show that intracellular Lat-A infusion into DG granule cells had no effect on LTP elicited by LPP stimulation (*n* = 7/group). ***C***, Field EPSP recordings of LPP responses show that extracellular Lat-A perfusion (500 nm, at horizontal bar) disrupted the stabilization of LPP-LTP (fEPSP recordings; *p* = 0.0002, *n* = 8/group).

We used Lat-A, which sequesters monomeric G-actin leading to the dissolution of recently formed, “treadmilling” actin polymers (Lam et al., 2015), to test the broad hypothesis described above. Because Lat-A interferes with cytoskeletal processes required for postsynaptic LTP (Kramár et al., 2006; Rex et al., 2007), we compared the effects of intracellular infusion of Lat-A to block postsynaptic actin dynamics with those of extracellular application, which acts both presynaptically and postsynaptically. Including 400 nm Lat-A in the clamp electrode, leading to intracellular infusion of the toxin, depressed LTP in CA1 pyramidal cells by >50% (veh, 125.8 ± 19.5%; Lat-A, 56.9 ± 16.5%; *p* < 0.026) without disturbing baseline physiology (Fig. 5A). This result corroborates prior reports that LTP in field CA1 depends on postsynaptic actin polymerization; it also provides evidence that intracellular infusion reaches the secondary and tertiary branches on which synaptic modifications occur. In contrast, clamp electrode infusion of the same concentration of Lat-A into granule cells had no effect on the magnitude of LPP-LTP (control, 141.7 ± 24.0% LTP; Lat-A, 145.6 ± 23.8%; Fig. 5B). However, extracellular applications of Lat-A significantly reduced LPP potentiation (veh 36.9 ± 3.4%; Lat-A, 16.7 ± 2.1%; *p* = 0.0002; Fig. 5C). These results indicate that the expression of endocannabinoid-dependent LTP in the LPP requires adjustments to the presynaptic actin cytoskeleton.

### Contributions of endocannabinoid-dependent LPP-LTP to memory

**Figure 6.**
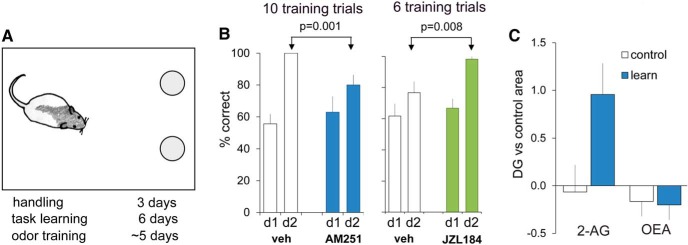
Odor learning engages, and depends upon, elements of LPP-LTP. ***A***, Rats were trained to find rewards in two small cups and then were given a series of novel two-odor discriminations with experiments conducted on the final discrimination. ***B***, Percentage of correct responses for the first 5 of 10 trials on the final discrimination (d1, day 1) and then on retention trials administered 24 h later (d2, day 2; no drug present). ***B***, Left, Rats injected with veh before training (open bars) had a high percentage of correct scores on d2, while rats injected with AM251 (1 mg/kg; filled bars) did not (*p* = 0.001, *t* test; *n* = 10/group). Right, Rats were given only six discrimination trials on d1 and then were retested for retention 24 h later: animals given the MGL inhibitor JZL184 (16 mg/kg) before d1 training had a higher percentage of correct scores on d2 than did vehicle-treated rats (*p* = 0.008; *n* = 10/group). ***C***, Rats in the experimental (learn) group (*n* = 15) were trained (10 trials) on odor cues, and then the DG was assayed for levels of 2-AG and the related lipid OEA; values were normalized within animals to those from auditory cortex or field CA1 (used as comparison regions in separate cohorts) and then were converted to *z*-scores (the number of SDs from the control group mean for each individual from each group). Control rats (*n* = 13) were transported to, but not placed in, the testing environment. DG 2-AG levels were significantly greater in the trained (“learn”) group than in control rats (*p* = 0.028, unpaired, two-tailed *t* test); OEA levels were not different between groups.

The LPP serves as the gateway for olfactory and associational information from piriform and polymodal regions of neocortex, respectively (Insausti et al., 1987; Burwell, 2000; Witter et al., 2000). Consonant with this, past studies showed that lesions of the LPP severely impair the formation of memory for simultaneous (but not serial) two-odor discriminations (Stäubli et al., 1984; Otto et al., 1991). We tested whether pharmacological manipulation of 2-AG signaling disturbs this form of odor learning, as would be expected if LPP-LTP were involved. Rats were trained on a series of simultaneous two-odor discriminations, each involving a different odor pair (one pair presented in 10 trials/d; Fig. 6A). They were then tested for their ability to learn a new odor pair with the 10 training trials initiated 1 h after the injection of vehicle or AM251 (1 mg/kg, i.p.): the percentage of correct responses was assessed for the first five trials during this final discrimination training (day 1) and during retention testing on day 5 (no drug) trials 24 h later (day 2). Vehicle-treated rats showed clear retention, with scores on day 2 being comparable to those previously described for this paradigm (Larson et al., 1995; Martens et al., 2013), whereas the AM251 group did not (Fig. 6B, left; *p* = 0.001, two-tailed *t* test). Because CB_1_ has been implicated in processing olfactory signals (Soria-Gómez et al., 2014), we repeated the comparisons using only those rats that exhibited strong evidence for learning on day 1 (≥80% correct on trials 6–10). Again, we found a marked difference in retention between control and AM251-treated rats (*p* = 0.018; Fig. 6B, right), suggesting that CB_1_ blockade impaired the formation of long-term memory in rats that had initially acquired the odor discrimination.

As described above, elevating 2-AG levels with the MGL inhibitor JZL184 markedly enhanced LTP in the LPP (Fig. 2C). We tested whether the compound (16 mg/kg) given 30 min before odor discrimination training also strengthens the encoding of long-term memory. Six training trials on day 1 were not sufficient to produce high retention scores in tests administered 24 h later for vehicle-treated rats but resulted in clear evidence for long-term memory in the JZL184 treatment group (*p* = 0.008; Fig. 6B, right).

The above results suggest that LPP-dependent learning initiates 2-AG signaling in the DG. To test this idea, we subjected rats to the day 1, 10 trial training protocol, snap froze their brains, and measured 2-AG content in lipid extracts using liquid chromatography/mass spectrometry (Jung et al., 2012a). Dentate gyrus 2-AG levels were significantly elevated in trained rats, compared with untrained controls (*p* = 0.028, *t* test; Fig. 6C). Anandamide levels were undetectable in both trained and control rats, and no group differences were evident for other endocannabinoid-related lipids, such as oleoylethanolamide (Fig. 6C).

## Discussion

**Figure 7.**
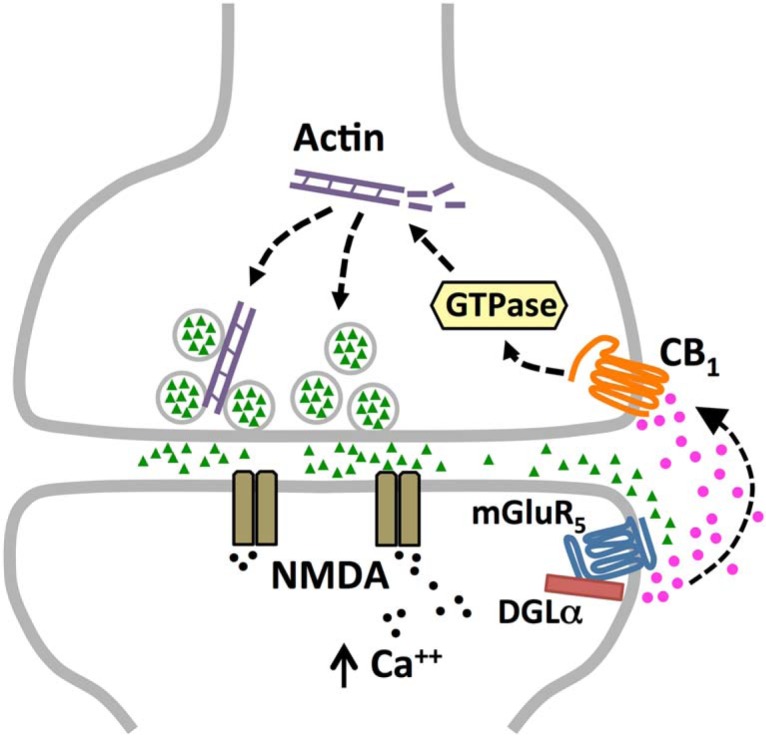
Schematic illustration of the proposed substrates for LTP in the LPP. The present evidence, and results described in the literature, suggest that the illustrated events are critical for the production of LPP-LTP. Glutamate (green triangles) released with brief bursts of high-frequency afferent stimulation activates NMDA- and mGluR5-type glutamate receptors and increases postsynaptic calcium content; these events engage the 2-AG synthesizing enzyme DGL-α. 2-AG produced postsynaptically then diffuses across the synapse and binds to CB_1_ receptors on LPP terminals, thereby initiating signaling via small GTPases and the assembly of latrunculin-sensitive actin filaments. The resultant reorganization of the terminal actin cytoskeleton enhances evoked transmitter release.

In many central synapses, dendritic spine-derived 2-AG engages CB_1_ receptors on axon terminals to cause short-term or long-term depression of neurotransmitter release (Kano et al., 2009; Castillo et al., 2012). The present results extend this standard model by showing that 2-AG-dependent retrograde signaling mediates synaptic potentiation, rather than inhibition, at the glutamatergic synapses formed by the LPP with granule cells of the DG. As schematized in Figure 7, the results indicate that this form of endocannabinoid-mediated LTP is initiated postsynaptically through a mechanism that requires the activation of mGluR5 and NMDA receptors along with increases in postsynaptic calcium, to influence DGL-α and the production of 2-AG, but is expressed in axon terminals via a long-lasting increase in glutamate release. Experiments with a selective toxin that prevents actin filament assembly (latrunculin A) provided evidence that the latter effect involves reorganization of the actin cytoskeleton in LPP terminals. Activation of CB_1_ receptors by 2-AG plays an obligatory, initiating role in shifting the boutons into the potentiated state.

Using pharmacological and genetic manipulation of 2-AG content and CB_1_ receptor function, and electrophysiological analysis of hippocampal slices, we found no evidence for a 2-AG contribution to LTP at sites outside the LPP (i.e., MPP, CA1), although earlier studies have described both positive and negative effects of CB_1_-related compounds on induction in other systems (see Introduction). The present results were obtained with near-threshold parameters for inducing robust and stable LTP, and it is possible that a modulatory influence of endocannabinoids emerges with different conditions at various locations in hippocampus. Pertinent to this, a recent report (Xu et al., 2012) described a presynaptic, endocannabinoid-dependent form of LTP in the S-C afferents of field CA1 elicited with paired stimulation of S-C and perforant path axons. Unlike the case for the LPP described here, this paired stimulation CA1 effect was dependent upon GABAergic transmission. Another group identified a novel, heterosynaptic form of presynaptic LTP in the S-C system triggered by 2-AG acting on astrocytes (Navarrete and Araque, 2010; Gómez-Gonzalo et al., 2015). Given that LTP production in the S-C projections by minimal TB stimulation (Larson et al., 1986; Larson and Lynch, 1989) is synapse specific, intact in CB_1_ knockouts (Fig. 1) and expressed postsynaptically (Muller and Lynch, 1988; Manabe and Nicoll, 1994; Kerchner and Nicoll, 2008; Granger and Nicoll, 2014), these recent findings raise the intriguing possibility that S-C synapses have a normally unused capacity to express a form of potentiation of a kind described here for the LPP. Possibly related to this, the present studies compared LPP results obtained from whole-cell recording with those from the less invasive field recording. It may be useful to use similar comparisons in future studies of other connections as clamp electrodes are known to affect channel operations and receptor kinetics (Mody et al., 1988; Simmons and Schneider, 1998; Lin et al., 2002).

Further differentiation of the LTP-related role of endocannabinoids in the LPP versus the S-C system was obtained using the CB_1_ agonist WIN. Past studies showed that WIN treatment potently impairs S-C LTP (Paton et al., 1998), whereas we found that WIN enhances potentiation in the LPP. It is noteworthy that fast gamma activity (65-130 Hz) occurs in the entorhinal cortex of behaving animals and extends across multiple cycles of the theta rhythm (Colgin et al., 2009); thus ∼100 Hz activity (as used for the production of LPP-LTP) is present for hundreds of milliseconds in the perforant path. The near-threshold LPP stimulation used in the WIN treatment studies is not greatly different from this pattern.

The failure to detect endocannabinoid-initiated, presynaptic LTP in MPP terminals, which innervate the granule cells in the field immediately adjacent to the LPP, was somewhat surprising. Little is known about the substrates for LTP expression in the MPP, although the available evidence points to postsynaptic modifications (Bramham, 2008; Harney et al., 2008). As noted, the mossy fibers generated by the granule cells are reported to be unresponsive to agents that affect CB_1_ receptors (Hofmann et al., 2008). These findings reinforce the conclusion that conventional stimulation protocols do not elicit the type of LTP found in the LPP at other sites in hippocampus.

An intriguing feature of potentiation in the LPP is that the magnitude of the effect, as recorded under control conditions, is much less than maximal: the inhibition of the 2-AG hydrolyzing enzyme MGL nearly doubled the magnitude of LPP potentiation. Moreover, in comparison with measures in field recording experiments, LPP-LTP was substantially larger in voltage-clamp experiments in which induction occurs under circumstances that maximize the opening of NMDAR channels. This pattern of results suggests that in response to threshold levels of stimulation some individual LPP terminals do not fully potentiate or that a large subpopulation of activated contacts fails to shift into the potentiated state. Notably, the difference between extracellular versus clamp recording with regard to LTP-related decreases in PPF were not comparable to the marked separation in the magnitude of LTP. This argues against the possibility that the large-amplitude LPP-LTP observed in whole-cell preparations was due to greater release from individual terminals in comparison to that in field potential studies. Instead, the results from MGL inhibition and clamp recording experiments point to the hypothesis that NMDAR activation at some LPP contacts was not sufficient to generate the 2-AG signaling that triggers the presynaptic expression of potentiation in this system. This idea finds precedent in studies of postsynaptic LTP in field CA1, where approximately half the synapses were shown to have high plasticity thresholds (Kramár et al., 2012; Lynch et al., 2013).

Studies of postsynaptic LTP have also provided considerable information about the mechanisms that express and stabilize the potentiated state. These include membrane trophic (BDNF; Korte et al., 1998; Panja and Bramham, 2014), steroid (estradiol; Kramár et al., 2009), and adhesion (Kramár et al., 2006; Bozdagi et al., 2010; Babayan et al., 2012) receptors that engage multiple actin signaling pathways that reorganize the subsynaptic cytoskeleton (Seese et al., 2012; Lynch et al., 2013; Rudy, 2015). Given the present evidence that LPP-LTP depends on presynaptic actin filament assembly, it is conceivable that comparable events within LPP axon terminals mediate the production of this endocannabinoid-dependent potentiation. Pertinent to this, recent studies have described novel endocannabinoid signaling in pancreatic cells (Malenczyk et al., 2013) and neuronal cell lines (Derkinderen et al., 2001; Dalton et al., 2013; Roland et al., 2014; Mai et al., 2015; Njoo et al., 2015) that involves GTPase and other actin regulatory cascades, interactions with surface adhesion receptors belonging to the integrin family, and cytoskeletal reorganization. There is an extensive body of work showing that actin networks in axon terminals influence the docking of transmitter vesicles and, thus, levels of evoked release (Sankaranarayanan et al., 2003; Park and Loh, 2008). It will be of considerable interest in future work to test whether the assays and manipulations used to identify cytoskeletal substrates of postsynaptic LTP detect comparable presynaptic events associated with endocannabinoid-dependent LPP-LTP. Such studies would require the broad range of technologies used in the analysis of stabilization mechanisms in field CA1.

The results of several studies indicate that endocannabinoids are involved in memory formation (Hampson et al., 2011; Ratano et al., 2014; Tan et al., 2014; Atsak et al., 2015; Goodman and Packard, 2015; Santana et al., 2016), and we confirmed the prediction from LTP experiments that manipulations of 2-AG signaling that were shown here to suppress or enhance LPP-LTP produce corresponding effects on LPP-dependent learning. Specifically, we found that these manipulations had predicted effects on odor discrimination learning. These behavioral results were obtained using peripheral injections of the compounds (AM251, JZL184), and thus it is possible that global effects could have contributed to the observed changes in memory encoding. Arguing against this is the observation that drug-treated animals acquired the discrimination during day 1 testing, indicating that cue processing was intact. More detailed analyses will be possible if further slice studies identify actin signaling events required for the stabilization of LPP-LTP, and if these markers prove applicable to behavioral studies (as was the case for field CA1; Fedulov et al., 2007; Chen et al., 2010; Seese et al., 2014). Local applications of CB_1_ and 2-AG-related compounds could then be used to target the DG to further test the role of endocannabinoids, and LPP-LTP, in memory encoding.

A substantial literature indicates that conventional, postsynaptic LTP, as best characterized in field CA1, provides a substrate for several types of memory (Morris, 2003; Sigurdsson et al., 2007; Nabavi et al., 2014; Rudy, 2015), and so the question arises, why is this type of plasticity not used by the LPP? And, related to this, what is the functional significance of utilizing distinct forms of plasticity in the two cortical inputs to the dentate gyrus? One possibility relates to the strong likelihood that the two afferents, MPP and LPP, convey different, simultaneously present categories of information. Recent behavioral and imaging studies led to the hypothesis that the MPP and LPP play complementary roles in the encoding of the “what,” “when,” and “where” components of episodic memories, with the MPP related to spatial relationships between cues (where) and the LPP to cue identity (what; Reagh and Yassa, 2014). Individual granule cells are innervated by both systems and then project to the pyramidal cells of the hippocampus proper where a third dimension—temporal order—is added to the memory of a cue sequence (Eichenbaum, 2014). It is possible that granule cells separate the two types of information they receive by encoding one and then the other on successive steps in a cycle, a process that would be facilitated by introducing a specialized form of plasticity at only one of the inputs. Pertinent to this, the dentate gyrus receives a small but functionally potent cholinergic projection originating in the medial septum/diagonal bands complex (MS/DBB; Amaral and Lavenex, 2007). Released acetylcholine both stimulates the synthesis of 2-AG (Kim et al., 2002) and depresses glutamate release from the MPP via a CB_1_-dependent mechanism (Colgin et al., 2003). It is therefore possible that elevated activity in the cholinergic inputs to the dentate gyrus could at the same time promote LTP in the LPP, via mechanisms described here, while disrupting potentiation in the MPP. In this sense, the MS/DBB would act as a gatekeeper that allows first one and then a second type of information to be encoded on the same neurons.

## Synthesis

The decision was a result of the Reviewing Editor Howard Eichenbaum and the peer reviewers coming together and discussing their recommendations until a consensus was reached. A fact-based synthesis statement explaining their decision and outlining what is needed to prepare a revision is listed below. The following reviewers agreed to reveal their identity: Pablo Castillo, Vijayalakshmi Santhakumar

While the authors have addressed some of the concerns in the original submission, and one reviewer is satisfied, the other reviewer has some remaining concerns that must be addressed.

Reviewer #1:

In this revised manuscript, the authors have addressed some of my previous concerns. Specifically, they have modified the text and performed one of the several experiments I requested. Important points remain unanswered, and there are still a number of loose ends regarding the mechanism underlying ecLTP.

1) In my previous report I requested: "The authors must test the prediction that a CB1 agonist potentiates LPP (but not MPP) synaptic transmission, in a FAK signaling-dependent but GABAa receptor-independent manner." The idea here is to test whether activation of CB1 receptors is sufficient to mimic ecLTP. The authors decided not to perform this simple experiment. Instead, they report that the CB1 receptor agonist WIN facilitates induction by some unknown mechanism. Moreover, it is unclear whether inhibition was intact in the new experiments reported in Fig 2J. In lines 353/354, they also claim that WIN had no evident effect on baseline responses. However, it appears that WIN actually increases synaptic transmission at the end of the bath application, right before the LTP induction protocol (Fig 2J). They should keep recording baseline transmission for two hours in the absence of the LTP induction protocol. Regarding the mechanism of CB1 receptor-mediated potentiation, the authors propose that FAK signaling could be involved but decided not to explore this possibility experimentally. It is unclear why they are reluctant to test FAK inhibitors.

2) "The presynaptic localization of CB1 receptors in LPP terminals is not convincing." Additional evidence that CB1 receptors colocalize with some specific marker of glutamatergic terminals is required (see Uchigashima et al, 2011, J. Neurosci 31:7700 -7714). In the absence of such marker(s), or LPP tracers as well suggested by reviewer #1, the results reported in Fig 4AB are not conclusive and should be removed. How do we know that the image in Fig 4A is not from a GABAergic terminal? The authors should show STORM images of CB1/PSD95 distribution as in 4A.

3) Remove from the text (and figure legend) all suggestions that ecLTP contributes to memory. The study does not provide any direct evidence in support of this link. The results are, at best, correlational.

4) The authors must provide a model (cartoon) of how ecLTP works. For example, the model should indicate whether NMDARs and mGluRs are pre or postsynaptic, how they are activated and how they engage endocannabinoid signaling at LPP-DGC synapses.

5) Given that LPP-LTP was blocked by postsynaptic BAPTA and d-APV (Fig. 3A,B), can the authors discard the contribution of a Hebbian component? Is LPP-LTP abolished in CB1 KO mice? Is it impaired by MPEP and extracellular Lat-A? These points have not been addressed in the revised manuscript. The authors only indicate that SR141716A dramatically reduced HFS-induced LTP (Fig 1A, inset). They assume, but have not actually tested, whether HFS-induced LTP and pairing protocol-induced LTP are mechanistically identical.

6) To my question "Why vehicle applications increase baseline transmission in DG but not CA1?", the authors responded: "The only case in which vehicle infusion produced an evident increase in baseline involved the compound WWL70". However, clear changes in baseline can also be observed in Fig. 1A, 1B, 2C, 2G, making the interpretation and quantification of the results rather difficult.

7) Re: presynaptic expression of ecLTP, the authors may want to add some qualifier throughout the text and modify their conclusions as follows "LTP in the LPP is *likely* expressed presynaptically".

8) If the authors want to claim that ecLTP is input-specific -i.e. expressed at LPP but not MPP synapses- they should test whether the exact induction protocols that trigger ecLTP at LPP synapses do not induce ecLTP at MPP synapses.

9) Fig 1A inset: show time course plot as for all other LTP plots in this figure.

## References

[B1] Abraham WC, Christie BR, Logan B, Lawlor P, Dragunow M (1994) Immediate early gene expression associated with the persistence of heterosynaptic long-term depression in the hippocampus. Proc Natl Acad Sci U S A 91:10049–10053. 7937835 10.1073/pnas.91.21.10049 PMC44955

[B2] Amaral D, Lavenex P (2007) Hippocampal neuroanatomy In: The hippocampus book (P Andersen, RG Morris, D Amaral, TV Bliss, J O'Keefe, eds), pp 37–114. New York: Oxford UP.

[B3] Atsak P, Hauer D, Campolongo P, Schelling G, Fornari RV, Roozendaal B (2015) Endocannabinoid signaling integrates multiple stress hormone effects on memory consolidation. Psychoneuroendocrinology 61:5. 10.1016/j.psyneuen.2015.07.400 25547713 PMC4397407

[B4] Babayan AH, Kramár EA, Barrett RM, Jafari M, Häettig J, Chen LY, Rex CS, Lauterborn JC, Wood MA, Gall CM, Lynch G (2012) Integrin dynamics produce a delayed stage of long-term potentiation and memory consolidation. J Neurosci 32:12854–12861. 10.1523/JNEUROSCI.2024-12.2012 22973009 PMC3752079

[B5] Bisogno T, Howell F, Williams G, Minassi A, Cascio MG, Ligresti A, Matias I, Schiano-Moriello A, Paul P, Williams EJ, Gangadharan U, Hobbs C, Di Marzo V, Doherty P (2003) Cloning of the first sn1-DAG lipases points to the spatial and temporal regulation of endocannabinoid signaling in the brain. J Cell Biol 163:463–468. 10.1083/jcb.200305129 14610053 PMC2173631

[B6] Bohme GA, Laville M, Ledent C, Parmentier M, Imperato A (2000) Enhanced long-term potentiation in mice lacking cannabinoid CB1 receptors. Neuroscience 95:5–7. 10619457 10.1016/s0306-4522(99)00483-2

[B7] Bozdagi O, Wang XB, Nikitczuk JS, Anderson TR, Bloss EB, Radice GL, Zhou Q, Benson DL, Huntley GW (2010) Persistence of coordinated long-term potentiation and dendritic spine enlargement at mature hippocampal CA1 synapses requires N-cadherin. J Neurosci 30:9984–9989. 10.1523/JNEUROSCI.1223-10.2010 20668183 PMC2921177

[B8] Bramham CR (2008) Local protein synthesis, actin dynamics, and LTP consolidation. Curr Opin Neurobiol 18:524–531. 10.1016/j.conb.2008.09.013 18834940

[B9] Bramham CR, Errington ML, Bliss TV (1988) Naloxone blocks the induction of long-term potentiation in the lateral but not in the medial perforant pathway in the anesthetized rat. Brain Res 449:352–356. 3395853 10.1016/0006-8993(88)91052-9

[B10] Burwell RD (2000) The parahippocampal region: corticocortical connectivity. Ann N Y Acad Sci 911:25–42. 10911865 10.1111/j.1749-6632.2000.tb06717.x

[B11] Carlson G, Wang Y, Alger BE (2002) Endocannabinoids facilitate the induction of LTP in the hippocampus. Nat Neurosci 5:723–724. 10.1038/nn879 12080342

[B12] Castillo PE, Younts TJ, Chávez AE, Hashimotodani Y (2012) Endocannabinoid signaling and synaptic function. Neuron 76:70–81. 10.1016/j.neuron.2012.09.020 23040807 PMC3517813

[B13] Chen LY, Rex CS, Sanaiha Y, Lynch G, Gall CM (2010) Learning induces neurotrophin signaling at hippocampal synapses. Proc Natl Acad Sci U S A 107:7030–7035. 10.1073/pnas.0912973107 20356829 PMC2872439

[B14] Chiu CQ, Castillo PE (2008) Input-specific plasticity at excitatory synapses mediated by endocannabinoids in the dentate gyrus. Neuropharmacology 54:68–78. 10.1016/j.neuropharm.2007.06.026 17706254 PMC2225485

[B15] Christie BR, Abraham WC (1994) Differential regulation of paired-pulse plasticity following LTP in the dentate gyrus. Neuroreport 5:385–388. 8003660 10.1097/00001756-199401120-00003

[B16] Colgin LL, Kramár EA, Gall CM, Lynch G (2003) Septal modulation of excitatory transmission in hippocampus. J Neurophysiol 90:2358–2366. 10.1152/jn.00262.2003 12840078

[B17] Colgin LL, Denninger T, Fyhn M, Hafting T, Bonnevie T, Jensen O, Moser MB, Moser EI (2009) Frequency of gamma oscillations routes flow of information in the hippocampus. Nature 462:353–357. 10.1038/nature08573 19924214

[B18] Cui Y, Paillé V, Xu H, Genet S, Delord B, Fino E, Berry H, Venance L (2015) Endocannabinoids mediate bidirectional striatal spike-timing-dependent plasticity. J Physiol 593:2833–2849. 10.1113/JP270324 25873197 PMC4506184

[B19] Dalton GD, Peterson LJ, Howlett AC (2013) CB_1_ cannabinoid receptors promote maximal FAK catalytic activity by stimulating cooperative signaling between receptor tyrosine kinases and integrins in neuronal cells. Cell Signal 25:1665–1677. 10.1016/j.cellsig.2013.03.020 23571270 PMC4165595

[B20] Dani A, Huang B, Bergan J, Dulac C, Zhuang X (2010) Superresolution imaging of chemical synapses in the brain. Neuron 68:843–856. 10.1016/j.neuron.2010.11.021 21144999 PMC3057101

[B21] Deng L, Cornett BL, Mackie K, Hohmann AG (2015) CB1 knockout mice unveil sustained CB2-mediated antiallodynic effects of the mixed CB1/CB2 agonist CP55,940 in a mouse model of paclitaxel-induced neuropathic pain. Mol Pharmacol 88:64–74. 10.1124/mol.115.098483 25904556 PMC4468646

[B22] Derkinderen P, Toutant M, Kadaré G, Ledent C, Parmentier M, Girault JA (2001) Dual role of Fyn in the regulation of FAK+6,7 by cannabinoids in hippocampus. J Biol Chem 276:38289–38296. 10.1074/jbc.M105630200 11468287

[B23] Do VH, Martinez CO, Martinez JL Jr, Derrick BE (2002) Long-term potentiation in direct perforant path projections to the hippocampal CA3 region in vivo. J Neurophysiol 87:669–678. 11826036 10.1152/jn.00938.2000

[B24] Dunwiddie T, Lynch G (1978) Long-term potentiation and depression of synaptic responses in the rat hippocampus: localization and frequency dependency. J Physiol 276:353–367. 650459 10.1113/jphysiol.1978.sp012239 PMC1282430

[B25] Eichenbaum H (2014) Time cells in the hippocampus: a new dimension for mapping memories. Nat Rev Neurosci 15:732–744. 10.1038/nrn3827 25269553 PMC4348090

[B26] Fedulov V, Rex CS, Simmons DA, Palmer L, Gall CM, Lynch G (2007) Evidence that long-term potentiation occurs within individual hippocampal synapses during learning. J Neurosci 27:8031–8039. 10.1523/JNEUROSCI.2003-07.2007 17652593 PMC6672739

[B27] Gómez-Gonzalo M, Navarrete M, Perea G, Covelo A, Martín-Fernández M, Shigemoto R, Luján R, Araque A (2015) Endocannabinoids induce lateral long-term potentiation of transmitter release by stimulation of gliotransmission. Cereb Cortex 25:3699–3712. 10.1093/cercor/bhu231 25260706

[B28] Goodman J, Packard MG (2015) The influence of cannabinoids on learning and memory processes of the dorsal striatum. Neurobiol Learn Mem 125:1–14. 10.1016/j.nlm.2015.06.008 26092091

[B29] Granger AJ, Nicoll RA (2014) Expression mechanisms underlying long-term potentiation: a postsynaptic view, 10 years on. Philos Trans R Soc Lond B Biol Sci 369:20130136 10.1098/rstb.2013.0136 24298139 PMC3843869

[B30] Gulyas AI, Cravatt BF, Bracey MH, Dinh TP, Piomelli D, Boscia F, Freund TF (2004) Segregation of two endocannabinoid-hydrolyzing enzymes into pre- and postsynaptic compartments in the rat hippocampus, cerebellum and amygdala. Eur J Neurosci 20:441–458. 10.1111/j.1460-9568.2004.03428.x 15233753

[B31] Hampson RE, Sweatt AJ, Goonawardena AV, Song D, Chan RH, Marmarelis VZ, Berger TW, Deadwyler SA (2011) Memory encoding in hippocampal ensembles is negatively influenced by cannabinoid CB1 receptors. Behav Pharmacol 22:335–346. 10.1097/FBP.0b013e3283473bfd 21558844 PMC3135765

[B32] Hanse E, Gustafsson B (1992) Postsynaptic, but not presynaptic, activity controls the early time course of long-term potentiation in the dentate gyrus. J Neurosci 12:3226–3240. 1386624 10.1523/JNEUROSCI.12-08-03226.1992 PMC6575651

[B33] Harney SC, Jane DE, Anwyl R (2008) Extrasynaptic NR2D-containing NMDARs are recruited to the synapse during LTP of NMDAR-EPSCs. J Neurosci 28:11685–11694. 10.1523/JNEUROSCI.3035-08.2008 18987204 PMC3844786

[B34] He JC, Gomes I, Nguyen T, Jayaram G, Ram PT, Devi LA, Iyengar R (2005) The G alpha(o/i)-coupled cannabinoid receptor-mediated neurite outgrowth involves Rap regulation of Src and Stat3. J Biol Chem 280:33426–33434. 10.1074/jbc.M502812200 16046413

[B35] Hofmann ME, Nahir B, Frazier CJ (2008) Excitatory afferents to CA3 pyramidal cells display differential sensitivity to CB1 dependent inhibition of synaptic transmission. Neuropharmacology 55:1140–1146. 10.1016/j.neuropharm.2008.07.007 18675282 PMC2610849

[B36] Howlett AC, Mukhopadhyay S (2000) Cellular signal transduction by anandamide and 2-arachidonoylglycerol. Chem Phys Lipids 108:53–70. 11106782 10.1016/s0009-3084(00)00187-0

[B37] Insausti R, Amaral DG, Cowan WM (1987) The entorhinal cortex of the monkey: II. Cortical afferents. J Comp Neur 264:356–395. 10.1002/cne.902640306 2445796

[B38] Iversen LL (2008) The science of marijuana. New York: Oxford UP.

[B39] Jia Y, Gall CM, Lynch G (2010) Presynaptic BDNF promotes postsynaptic long-term potentiation in the dorsal striatum. J Neurosci 30:14440–14445. 10.1523/JNEUROSCI.3310-10.2010 20980601 PMC2972744

[B40] Jung KM, Mangieri R, Stapleton C, Kim J, Fegley D, Wallace M, Mackie K, Piomelli D (2005) Stimulation of endocannabinoid formation in brain slice cultures through activation of group I metabotropic glutamate receptors. Mol Pharmacol 68:1196–1202. 10.1124/mol.105.013961 16051747

[B41] Jung KM, Astarita G, Zhu C, Wallace M, Mackie K, Piomelli D (2007) A key role for diacylglycerol lipase-alpha in metabotropic glutamate receptor-dependent endocannabinoid mobilization. Mol Pharmacol 72:612–621. 10.1124/mol.107.037796 17584991

[B42] Jung KM, Sepers M, Henstridge CM, Lassalle O, Neuhofer D, Martin H, Ginger M, Frick A, DiPatrizio NV, Mackie K, Katona I, Piomelli D, Manzoni OJ (2012a) Uncoupling of the endocannabinoid signalling complex in a mouse model of fragile X syndrome. Nat Commun 3:1080 10.1038/ncomms2045 23011134 PMC3657999

[B43] Jung KM, Clapper JR, Fu J, D'Agostino G, Guijarro A, Thongkham D, Avanesian A, Astarita G, DiPatrizio NV, Frontini A, Cinti S, Diano S, Piomelli D (2012b) 2-Arachidonoylglycerol signaling in forebrain regulates systemic energy metabolism. Cell Metab 15:299–310. 10.1016/j.cmet.2012.01.021 22405068 PMC3729112

[B44] Kano M, Ohno-Shosaku T, Hashimotodani Y, Uchigashima M, Watanabe M (2009) Endocannabinoid-mediated control of synaptic transmission. Physiol Rev 89:309–380. 10.1152/physrev.00019.2008 19126760

[B45] Katona I, Urbán GM, Wallace M, Ledent C, Jung KM, Piomelli D, Mackie K, Freund TF (2006) Molecular composition of the endocannabinoid system at glutamatergic synapses. J Neurosci 26:5628–5637. 10.1523/JNEUROSCI.0309-06.2006 16723519 PMC1698282

[B46] Kauer JA, Malenka RC, Nicoll RA (1988) A persistent postsynaptic modification mediates long-term potentiation in the hippocampus. Neuron 1:911–917. 2908443 10.1016/0896-6273(88)90148-1

[B47] Keimpema E, Mackie K, Harkany T (2011) Molecular model of cannabis sensitivity in developing neuronal circuits. Trends Pharmacol Sci 32:551–561. 10.1016/j.tips.2011.05.004 21757242 PMC3159827

[B48] Kerchner GA, Nicoll RA (2008) Silent synapses and the emergence of a postsynaptic mechanism for LTP. Nat Rev Neurosci 9:813–825. 10.1038/nrn2501 18854855 PMC2819160

[B49] Khaspekov LG, Brenz Verca MS, Frumkina LE, Hermann H, Marsicano G, Lutz B (2004) Involvement of brain-derived neurotrophic factor in cannabinoid receptor-dependent protection against excitotoxicity. Eur J Neurosci 19:1691–1698. 10.1111/j.1460-9568.2004.03285.x 15078543

[B114] Kim J, Isokawa M, Ledent C, Alger BE (2002) Activation of muscarinic acetylcholine receptors enhances the release of endogenous cannabinoids in the hippocampus. J Neurosci 22:10182–10191. 12451119 10.1523/JNEUROSCI.22-23-10182.2002 PMC6758770

[B50] King AR, Duranti A, Tontini A, Rivara S, Rosengarth A, Clapper JR, Astarita G, Geaga JA, Luecke H, Mor M, Tarzia G, Piomelli D (2007) URB602 inhibits monoacylglycerol lipase and selectively blocks 2-arachidonoylglycerol degradation in intact brain slices. Chem Biol 14:1357–1365. 10.1016/j.chembiol.2007.10.017 18096504 PMC2225625

[B51] Korte M, Kang H, Bonhoeffer T, Schuman E (1998) A role for BDNF in the late-phase of hippocampal long-term potentiation. Neuropharmacology 37:553–559. 9704996 10.1016/s0028-3908(98)00035-5

[B52] Kramár EA, Lin B, Rex CS, Gall CM, Lynch G (2006) Integrin-driven actin polymerization consolidates long-term potentiation. Proc Natl Acad Sci U S A 103:5579–5584. 10.1073/pnas.0601354103 16567651 PMC1459396

[B53] Kramár EA, Chen LY, Brandon NJ, Rex CS, Liu F, Gall CM, Lynch G (2009) Cytoskeletal changes underlie estrogen's acute effects on synaptic transmission and plasticity. J Neurosci 29:12982–12993. 10.1523/JNEUROSCI.3059-09.2009 19828812 PMC2806054

[B54] Kramár EA, Babayan AH, Gavin CF, Cox CD, Jafari M, Gall CM, Rumbaugh G, Lynch G (2012) Synaptic evidence for the efficacy of spaced learning. Proc Natl Acad Sci U S A 109:5121–5126. 10.1073/pnas.1120700109 22411798 PMC3323981

[B55] Lam PY, Mangos S, Green JM, Reiser J, Huttenlocher A (2015) In vivo imaging and characterization of actin microridges. PLoS One 10:e0115639. 10.1371/journal.pone.0115639 25629723 PMC4309568

[B56] Larson J, Lynch G (1989) Theta pattern stimulation and the induction of LTP: the sequence in which synapses are stimulated determines the degree to which they potentiate. Brain Res 489:49–58. 2743153 10.1016/0006-8993(89)90007-3

[B57] Larson J, Wong D, Lynch G (1986) Patterned stimulation at the theta frequency is optimal for the induction of hippocampal long-term potentiation. Brain Res 368:347–350. 3697730 10.1016/0006-8993(86)90579-2

[B58] Larson J, Lieu T, Petchpradub V, LeDuc B, Ngo H, Rogers GA, Lynch G (1995) Facilitation of olfactory learning by a modulator of AMPA receptors. J Neurosci 15:8023–8030. 8613739 10.1523/JNEUROSCI.15-12-08023.1995 PMC6577953

[B59] Lin B, Colgin LL, Brücher FA, Arai AC, Lynch G (2002) Interactions between recording technique and AMPA receptor modulators. Brain Res 955:164–173. 12419533 10.1016/s0006-8993(02)03429-7

[B60] Lin QS, Yang Q, Liu DD, Sun Z, Dang H, Liang J, Wang YX, Chen J, Li ST (2011) Hippocampal endocannabinoids play an important role in induction of long-term potentiation and regulation of contextual fear memory formation. Brain Res Bull 86:139–145. 10.1016/j.brainresbull.2011.07.011 21801815

[B61] Long JZ, Nomura DK, Cravatt BF (2009) Characterization of monoacylglycerol lipase inhibition reveals differences in central and peripheral endocannabinoid metabolism. Chem Biol 16:744–753. 10.1016/j.chembiol.2009.05.009 19635411 PMC2867454

[B62] Lovinger DM, Routtenberg A (1988) Synapse-specific protein kinase C activation enhances maintenance of long-term potentiation in rat hippocampus. J Physiol 400:321–333. 3418528 10.1113/jphysiol.1988.sp017122 PMC1191809

[B63] Lynch G, Gall CM (2013) Mechanism based approaches for rescuing and enhancing cognition. Front Neurosci 7:143. 10.3389/fnins.2013.00143 23966908 PMC3744010

[B64] Lynch G, Kramár EA, Babayan AH, Rumbaugh G, Gall CM (2013) Differences between synaptic plasticity thresholds result in new timing rules for maximizing long-term potentiation. Neuropharmacology 64:27–36. 10.1016/j.neuropharm.2012.07.006 22820276 PMC3445784

[B65] Mackie K (2008) Cannabinoid receptors: where they are and what they do. J Neuroendocrinol 20 [Suppl 1]:10–14. 10.1111/j.1365-2826.2008.01671.x 18426493

[B66] Mai P, Tian L, Yang L, Wang L, Yang L, Li L (2015) Cannabinoid receptor 1 but not 2 mediates macrophage phagocytosis by G(α)i/o/RhoA/ROCK signaling pathway. J Cell Physiol 230:1640–1650. 10.1002/jcp.24911 25545473

[B67] Malenczyk K, Jazurek M, Keimpema E, Silvestri C, Janikiewicz J, Mackie K, Di Marzo V, Redowicz MJ, Harkany T, Dobrzyn A (2013) CB1 cannabinoid receptors couple to focal adhesion kinase to control insulin release. J Biol Chem 288:32685–32699. 10.1074/jbc.M113.478354 24089517 PMC3820903

[B68] Manabe T, Nicoll RA (1994) Long-term potentiation: evidence against an increase in transmitter release probability in the CA1 region of the hippocampus. Science 265:1888–1892. 7916483 10.1126/science.7916483

[B69] Martens KM, Vonder Haar C, Hutsell BA, Hoane MR (2013) The dig task: a simple scent discrimination reveals deficits following frontal brain damage. J Vis Exp e50033. 10.3791/50033 PMC3582673 23328920

[B70] Mátyás F, Yanoversusky Y, Mackie K, Kelsch W, Misgeld U, Freund TF (2006) Subcellular localization of type 1 cannabinoid receptors in the rat basal ganglia. Neuroscience 137:337–361. 10.1016/j.neuroscience.2005.09.005 16289348

[B71] Mátyás F, Watanabe M, Mackie K, Katona I, Freund TF (2007) Molecular architecture of the cannabinoid signaling system in the core of the nucleus accumbens. Ideggyogy Sz 60:187–191. 17451066

[B72] Mody I, Salter MW, MacDonald JF (1988) Requirement of NMDA receptor/channels for intracellular high-energy phosphates and the extent of intraneuronal calcium buffering in cultured mouse hippocampal neurons. Neurosci Lett 93:73–78. 2850518 10.1016/0304-3940(88)90015-8

[B73] Moreno-Sanz G, Duranti A, Melzig L, Fiorelli C, Ruda GF, Colombano G, Mestichelli P, Sanchini S, Tontini A, Mor M, Bandiera T, Scarpelli R, Tarzia G, Piomelli D (2013) Synthesis and structure-activity relationship studies of O-biphenyl-3-yl carbamates as peripherally restricted fatty acid amide hydrolase inhibitors. J Med Chem 56:5917–5930. 10.1021/jm4007017 23822179 PMC4062305

[B74] Morris RG (2003) Long-term potentiation and memory. Philos Trans R Soc Lond B Biol Sci 358:643–647. 10.1098/rstb.2002.1230 12740109 PMC1693171

[B75] Muller D, Lynch G (1988) Long-term potentiation differentially affects two components of synaptic responses in hippocampus. Proc Natl Acad Sci U S A 85:9346–9350. 2904150 10.1073/pnas.85.23.9346 PMC282736

[B76] Nabavi S, Fox R, Proulx CD, Lin JY, Tsien RY, Malinow R (2014) Engineering a memory with LTD and LTP. Nature 511:348–352. 10.1038/nature13294 24896183 PMC4210354

[B77] Navarrete M, Araque A (2010) Endocannabinoids potentiate synaptic transmission through stimulation of astrocytes. Neuron 68:113–126. 10.1016/j.neuron.2010.08.043 20920795

[B78] Naydenov AV, Horne EA, Cheah CS, Swinney K, Hsu KL, Cao JK, Marrs WR, Blankman JL, Tu S, Cherry AE, Fung S, Wen A, Li W, Saporito MS, Selley DE, Cravatt BF, Oakley JC, Stella N (2014) ABHD6 blockade exerts antiepileptic activity in PTZ-induced seizures and in spontaneous seizures in R6/2 mice. Neuron 83:361–371. 10.1016/j.neuron.2014.06.030 25033180 PMC4136499

[B79] Nicoll RA, Schmitz D (2005) Synaptic plasticity at hippocampal mossy fibre synapses. Nat Rev Neurosci 6:863–876. 10.1038/nrn1786 16261180

[B80] Nithipatikom K, Gomez-Granados AD, Tang AT, Pfeiffer AW, Williams CL, Campbell WB (2012) Cannabinoid receptor type 1 (CB1) activation inhibits small GTPase RhoA activity and regulates motility of prostate carcinoma cells. Endocrinology 153:29–41. 10.1210/en.2011-1144 22087025 PMC3249681

[B81] Njoo C, Agarwal N, Lutz B, Kuner R (2015) The cannabinoid receptor CB1 interacts with the WAVE1 complex and plays a role in actin dynamics and structural plasticity in neurons. PLoS Biol 13:e1002286. 10.1371/journal.pbio.1002286 26496209 PMC4619884

[B82] Otto T, Schottler F, Staubli U, Eichenbaum H, Lynch G (1991) Hippocampus and olfactory discrimination learning: effects of entorhinal cortex lesions on olfactory learning and memory in a successive-cue, go-no-go task. Behav Neurosci 105:111–119. 10.1037/0735-7044.105.1.111 2025384

[B83] Panja D, Bramham CR (2014) BDNF mechanisms in late LTP formation: a synthesis and breakdown. Neuropharmacology 76:664–676. 10.1016/j.neuropharm.2013.06.024 23831365

[B84] Park JJ, Loh YP (2008) How peptide hormone vesicles are transported to the secretion site for exocytosis. Mol Endocrinol 22:2583–2595. 10.1210/me.2008-0209 18669645 PMC2626200

[B85] Paton GS, Pertwee RG, Davies SN (1998) Correlation between cannabinoid mediated effects on paired pulse depression and induction of long term potentiation in the rat hippocampal slice. Neuropharmacology 37:1123–1130. 9833642 10.1016/s0028-3908(98)00096-3

[B86] Piomelli D (2014) More surprises lying ahead. The endocannabinoids keep us guessing. Neuropharmacology 76:228–234. 10.1016/j.neuropharm.2013.07.026 23954677 PMC3855347

[B87] Ratano P, Everitt BJ, Milton AL (2014) The CB1 receptor antagonist AM251 impairs reconsolidation of pavlovian fear memory in the rat basolateral amygdala. Neuropsychopharmacology 39:2529–2537. 10.1038/npp.2014.103 24801769 PMC4149486

[B88] Reagh ZM, Yassa MA (2014) Object and spatial mnemonic interference differentially engage lateral and medial entorhinal cortex in humans. Proc Natl Acad Sci U S A 111:E4264–E4273. 10.1073/pnas.1411250111 25246569 PMC4210036

[B89] Rex CS, Lin CY, Kramár EA, Chen LY, Gall CM, Lynch G (2007) Brain-derived neurotrophic factor promotes long-term potentiation-related cytoskeletal changes in adult hippocampus. J Neurosci 27:3017–3029. 10.1523/JNEUROSCI.4037-06.2007 17360925 PMC6672589

[B90] Rodriguez A, Laio A (2014) Machine learning. Clustering by fast search and find of density peaks. Science 344:1492–1496. 10.1126/science.1242072 24970081

[B91] Roland AB, Ricobaraza A, Carrel D, Jordan BM, Rico F, Simon A, Humbert-Claude M, Ferrier J, McFadden MH, Scheuring S, Lenkei Z (2014) Cannabinoid-induced actomyosin contractility shapes neuronal morphology and growth. Elife 3:e03159. 10.7554/eLife.03159 25225054 PMC4179426

[B92] Rudy JW (2015) Actin dynamics and the evolution of the memory trace. Brain Res 1621:17–28. 10.1016/j.brainres.2014.12.007 25498985

[B93] Sankaranarayanan S, Atluri PP, Ryan TA (2003) Actin has a molecular scaffolding, not propulsive, role in presynaptic function. Nat Neurosci 6:127–135. 10.1038/nn1002 12536209

[B94] Santana F, Sierra RO, Haubrich J, Crestani AP, Duran JM, de Freitas Cassini L, de Oliveira Alvares L, Quillfeldt JA (2016) Involvement of the infralimbic cortex and CA1 hippocampal area in reconsolidation of a contextual fear memory through CB1 receptors: effects of CP55,940. Neurobiol Learn Mem 127:42–47. 10.1016/j.nlm.2015.11.016 26691779

[B95] Schmitz SK, King C, Kortleven C, Huson V, Kroon T, Kevenaar JT, Schut D, Saarloos I, Hoetjes JP, de Wit H, Stiedl O, Spijker S, Li KW, Mansvelder HD, Smit AB, Cornelisse LN, Verhage M, Toonen RF (2016) Presynaptic inhibition upon CB1 or mGlu2/3 receptor activation requires ERK/MAPK phosphorylation of Munc18-1. EMBO J 35:1236–1250. 10.15252/embj.201592244 27056679 PMC4888233

[B96] Seese RR, Babayan AH, Katz AM, Cox CD, Lauterborn JC, Lynch G, Gall CM (2012) LTP induction translocates cortactin at distant synapses in wild-type but not Fmr1 knock-out mice. J Neurosci 32:7403–7413. 10.1523/JNEUROSCI.0968-12.2012 22623686 PMC3365659

[B97] Seese RR, Wang K, Yao YQ, Lynch G, Gall CM (2014) Spaced training rescues memory and ERK1/2 signaling in fragile X syndrome model mice. Proc Natl Acad Sci U S A 111:16907–16912. 10.1073/pnas.1413335111 25385607 PMC4250145

[B98] Sigurdsson T, Doyère V, Cain CK, LeDoux JE (2007) Long-term potentiation in the amygdala: a cellular mechanism of fear learning and memory. Neuropharmacology 52:215–227. 10.1016/j.neuropharm.2006.06.022 16919687

[B99] Simmons MA, Schneider CR (1998) Regulation of M-type potassium current by intracellular nucleotide phosphates. J Neurosci 18:6254–6260. 9698318 10.1523/JNEUROSCI.18-16-06254.1998 PMC6793213

[B100] Soria-Gómez E, Bellocchio L, Reguero L, Lepousez G, Martin C, Bendahmane M, Ruehle S, Remmers F, Desprez T, Matias I, Wiesner T, Cannich A, Nissant A, Wadleigh A, Pape HC, Chiarlone AP, Quarta C, Verrier D, Vincent P, Massa F, Lutz B, Guzmán M, Gurden H, Ferreira G, Lledo PM, Grandes P, Marsicano G (2014) The endocannabinoid system controls food intake via olfactory processes. Nat Neurosci 17:407–415. 10.1038/nn.3647 24509429

[B101] Stäubli U, Ivy G, Lynch G (1984) Hippocampal denervation causes rapid forgetting of olfactory information in rats. Proc Natl Acad Sci U S A 81:5885–5887. 6592592 10.1073/pnas.81.18.5885 PMC391817

[B102] Stäubli U, Larson J, Lynch G (1990) Mossy fiber potentiation and long-term potentiation involve different expression mechanisms. Synapse 5:333–335. 10.1002/syn.890050410 2360200

[B103] Sugaya Y, Cagniard B, Yamazaki M, Sakimura K, Kano M (2013) The endocannabinoid 2-arachidonoylglycerol negatively regulates habituation by suppressing excitatory recurrent network activity and reducing long-term potentiation in the dentate gyrus. J Neurosci 33:3588–3601. 10.1523/JNEUROSCI.3141-12.2013 23426686 PMC6619537

[B104] Tan H, Ahmad T, Loureiro M, Zunder J, Laviolette SR (2014) The role of cannabinoid transmission in emotional memory formation: implications for addiction and schizophrenia. Front Psychiatry 5:73 10.3389/fpsyt.2014.00073 25071606 PMC4074769

[B105] Tart CT (1970) Marijuana intoxication common experiences. Nature 226:701–704. 5443246 10.1038/226701a0

[B106] Trieu BH, Kramár EA, Cox CD, Jia Y, Wang W, Gall CM, Lynch G (2015) Pronounced differences in signal processing and synaptic plasticity between piriform-hippocampal network stages: a prominent role for adenosine. J Physiol 593:2889–2907. 10.1113/JP270398 25902928 PMC4506187

[B107] Uchigashima M, Yamazaki M, Yamasaki M, Tanimura A, Sakimura K, Kano M, Watanabe M (2011) Molecular and morphological configuration for 2-arachidonoylglycerol-mediated retrograde signaling at mossy cell-granule cell synapses in the dentate gyrus. J Neurosci 31:7700–7714. 10.1523/JNEUROSCI.5665-10.2011 21613483 PMC6633146

[B108] van Groen T, Miettinen P, Kadish I (2003) The entorhinal cortex of the mouse: organization of the projection to the hippocampal formation. Hippocampus 13:133–149. 10.1002/hipo.10037 12625464

[B109] Wigström H, Gustafsson B (1986) Postsynaptic control of hippocampal long-term potentiation. J Physiol (Paris) 81:228–236. 2883309

[B110] Wilson DI, Watanabe S, Milner H, Ainge JA (2013) Lateral entorhinal cortex is necessary for associative but not nonassociative recognition memory. Hippocampus 23:1280–1290. 10.1002/hipo.22165 23836525 PMC4030623

[B111] Witter MP (1993) Organization of the entorhinal-hippocampal system: a review of current anatomical data. Hippocampus 3:33–44. 8287110

[B112] Witter MP, Wouterlood FG, Naber PA, Van Haeften T (2000) Anatomical organization of the parahippocampal-hippocampal network. Ann N Y Acad Sci 911:1–24. 10911864 10.1111/j.1749-6632.2000.tb06716.x

[B113] Xu JY, Zhang J, Chen C (2012) Long-lasting potentiation of hippocampal synaptic transmission by direct cortical input is mediated via endocannabinoids. J Physiol 590:2305–2315. 10.1113/jphysiol.2011.223511 22411015 PMC3424754

